# Inhibition of Receptor Dimerization as a Novel Negative Feedback Mechanism of EGFR Signaling

**DOI:** 10.1371/journal.pone.0139971

**Published:** 2015-10-14

**Authors:** Malgorzata Kluba, Yves Engelborghs, Johan Hofkens, Hideaki Mizuno

**Affiliations:** 1 Laboratory of Biomolecular Network Dynamics, Biochemistry, Molecular and Structural Biology Section, Department of Chemistry, KU Leuven, Celestijnenlaan 200G box 2403, 3001, Heverlee, Belgium; 2 Molecular Imaging and Photonics, Department of Chemistry, KU Leuven, Celestijnenlaan 200F, 3001, Heverlee, Belgium; Universidad del Pais Vasco, SPAIN

## Abstract

Dimerization of the epidermal growth factor receptor (EGFR) is crucial for initiating signal transduction. We employed raster image correlation spectroscopy to continuously monitor the EGFR monomer-dimer equilibrium in living cells. EGFR dimer formation upon addition of EGF showed oscillatory behavior with a periodicity of about 2.5 min, suggesting the presence of a negative feedback loop to monomerize the receptor. We demonstrated that monomerization of EGFR relies on phospholipase Cγ, protein kinase C, and protein kinase D (PKD), while being independent of Ca^2+^ signaling and endocytosis. Phosphorylation of the juxtamembrane threonine residues of EGFR (T654/T669) by PKD was identified as the factor that shifts the monomer-dimer equilibrium of ligand bound EGFR towards the monomeric state. The dimerization state of the receptor correlated with the activity of an extracellular signal-regulated kinase, downstream of the EGFR. Based on these observations, we propose a novel, negative feedback mechanism that regulates EGFR signaling via receptor monomerization.

## Introduction

**Fig 1:**
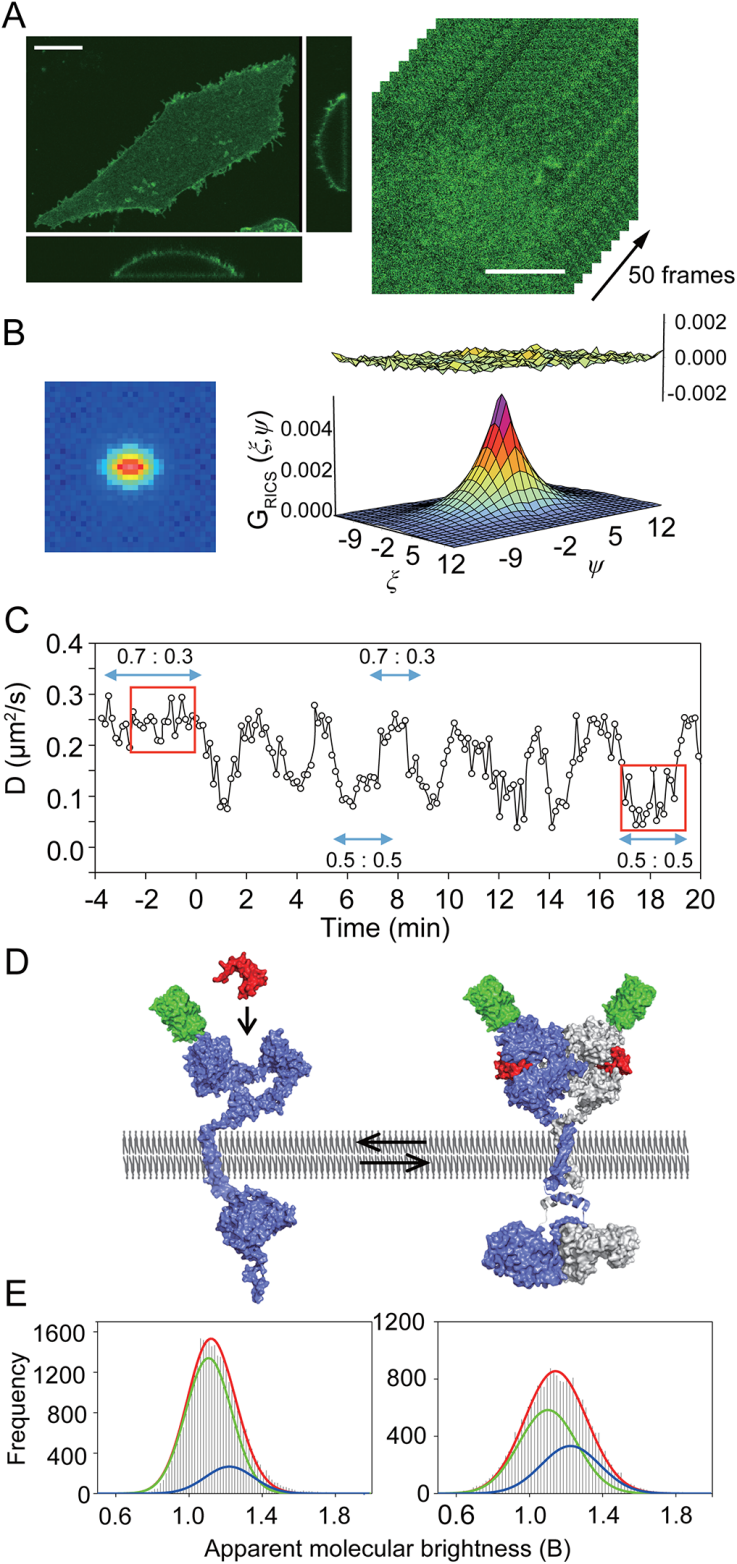
Repetitive change in the monomer to dimer ratio of EGFR revealed by RICS analysis. (A) (left) Plasma membrane localization of eGFP-EGFR^wt^ in CHO-K1 cells. Confocal images of the basal plasma membrane before EGF addition. The bottom and right panels show cross sections of the cell. The scale bar indicates 10 μm. (right) Example of 50 frames of eGFP-EGFR in CHO-K1 basal plasma membrane used to calculate the RICS auto-correlation. The scale bar indicates 5 μm. (B) RICS auto-correlation (left) of eGFP-EGFR^wt^ in CHO-K1, calculated for a time bin of 50 frames (for ξ and ψ from -16 to 16 pixels, respectively), fitted to the 2D diffusion model with residual plot (right). The residuals of the fitting being less than 10% of the magnitude of the function indicate the appropriate fit. (C) Reproducible time trace of eGFP-EGFR^wt^ diffusion coefficient. At the t = 0 EGF was added. Each point represents one time bin in RICS analysis, consisted of 50 frames which corresponds to 83 s, with 5 frame (8.3 s) interval. The EGFR monomer to dimer ratio at the regions indicated by arrows was calculated by N&B analysis. The experiment was repeated 20 times with essentially the same result. (D) Schematic structure of eGFP-EGFR monomer and dimer based on following PDB files: 3EVP, 1EGF, 2JWA, 1M17, 2M20, 2GS2 and 3GOP. EGF and eGFP are shown in red and green, respectively. 2JWA structure based on ErbB2 was used to display transmembrane helix dimer. (E) N&B analysis of eGFP-EGFR^wt^ before (left) and after (right) EGF addition (indicated with red boxes in C). Histograms of apparent molecular brightness are shown. Green and blue lines represent the distribution of the EGFR monomer (ε ≈ 0.1, B ≈ 1.1) and dimer (ε ≈ 0.2, B ≈ 1.2), respectively; red line shows the cumulative distribution of both monomer and dimer.

Epidermal growth factor receptor (EGFR) signaling plays a role in cell growth, differentiation, survival and proliferation [1]. The strength and duration of the signal are strictly controlled and dysregulation of this signaling pathway can lead to carcinogenesis [2–5]. EGFR consists of a ligand binding ectodomain followed by a transmembrane single-helix, a juxtamembrane (JM) segment and finally an intracellular kinase domain with a regulatory C-terminus (Fig 1D) [6, 7]. When epidermal growth factor (EGF) binds to the monomeric receptor, steric constraints are removed to expose the “dimerization arm” enabling the association of two EGFR units [8]. Subsequent association of the two transmembrane domains and an antiparallel interaction between the N-terminal juxtamembrane helices (JM-A) promotes EGFR activation [9]. The C-lobe of the kinase domain of one EGFR, the activator, interacts with the N-lobe of the binding partner’s kinase domain, the receiver, forming an asymmetric dimer [10]. Furthermore, the C-terminal juxtamembrane segment (JM-B) of the receiver forms a clamp over the C-lobe of the activator, stabilizing this interaction [9, 11]. EGFR kinase activity generates binding sites for downstream signaling proteins by transphosphorylating tyrosine residues in the C-terminus. Since the formation of the asymmetric dimer is critical for EGFR activation [10], its modulation might underlie a signaling regulation mechanism. However, regulatory mechanisms that affect the monomer-dimer equilibrium remain so far obscure. Mitogen-induced gene 6 (MIG6) is the only known inhibitor acting at the asymmetric dimer interface [12]. MIG6 is an inducible inhibitor, which requires *de novo* synthesis, therefore the feedback regulation by MIG6 takes place in the time range of an hour after EGF stimulation.

In general, diffusion coefficients of transmembrane proteins are inversely proportional to the radius of the membrane-crossing domain [13]. Single-particle tracking (SPT) of EGFR in living cells has revealed that a dimer with two intertwined transmembrane helices diffuses twice as slow as the monomeric form [14]. Although SPT is a critical tool for direct observation of protein dynamics and interactions [15], the number of molecules that can be examined at certain time period is limited, making it difficult to study rapid phenomena happening in the order of minute or faster. To circumvent this issue, we applied time-lapse raster image correlation spectroscopy (RICS) based on 2D spatial correlation analysis in raster-scanned images [16] to study time-dependency of EGFR dimerization in CHO cells. With this system, we observed oscillatory behavior of the monomer to dimer ratio of EGF-bound EGFR. This implied the presence of a negative feedback loop regulating dimer formation. By combining pharmacological and mutagenic approaches with time-lapse RICS analysis, we revealed a novel negative feedback mechanism.

## Results

### The EGFR monomer-dimer equilibrium shifts repetitively

We subcloned the eGFP-labeled EGFR (eGFP-EGFR^wt^) into a mammalian expression vector (eGFP-EGFR/pcDNA3) and expressed it transiently in CHO-K1 cells. In cells with a moderate fluorescent signal, eGFP-EGFR^wt^ was localized mainly in the plasma membrane (Fig 1A). However, in cells with very high expression level, surplus eGFP-EGFR accumulated in intracellular membranes. All measurements and analyses were performed on selected cells with moderate expression, in which eGFP-EGFR localized in the plasma membrane. The density of the receptors in the plasma membrane was calculated from the eGFP-EGFR fluorescence intensity in acquired images and found to be between 120–400 receptors/μm^2^ (S1 Fig). The average area of the basal plasma membrane of eGFP-EGFR transfected cells was calculated to 316±109 μm^2^ (n = 19). Assuming that the cell is thin enough to consider that both basal and apical cell membranes have equal surface area, we calculated the average surface area of the whole cell to be 632 μm^2^. From the area and the density of EGFR, the expression level was estimated to 0.7–2.5 × 10^5^ receptors/cell. This level was an order of magnitude lower as compared to A431 epidermoid carcinoma cells known for high EGFR expression. EGF binding was observed to the surface of CHO-K1 cells expressing eGFP-EGFR, whereas no EGF bound to non-transfected CHO-K1 cells (S2 Fig panel A). The functionality of eGFP-EGFR^wt^ was evaluated by observing Y1173 phosphorylation by immunostaining with an anti-pY1173 antibody (S2 Fig panel B) and EGF-mediated activation of downstream pathways, such as extracellular signal-regulated kinase (ERK) and Ca^2+^ in individual cells (S2 Fig panels D and E). During prolonged image acquisition, up to 20 min after EGF addition, eGFP-EGFR^wt^ fluorescence intensity slightly decreased (S2 Fig panel C), however the majority of EGFR stayed in the plasma membrane throughout the imaging duration.

RICS analysis was performed on the basal plasma membranes of cells that expressed eGFP-EGFR^wt^. The immobile fraction was filtered out using a moving average subtraction (S1 and S2 Movies). The mobile fraction was determined to 80 ± 12% by independent fluorescence recovery after photobleaching (FRAP) measurement. The EGFR diffusion coefficient (D_EGFR_^wt^) was determined by fitting the eGFP-EGFR autocorrelation to the 2D membrane diffusion model (Fig 1B). Under resting conditions, D_EGFR_^wt^ was 0.24 ± 0.02 μm^2^/s (Fig 1C), which is consistent with literary reported D_EGFR_ calculated using SPT [14]. We challenged the cells with a high concentration of EGF (0.17 μM) to ensure the activation of the pathways which require low-affinity interactions [17]. One minute after EGF addition, D_EGFR_^wt^ had decreased to 0.09 ± 0.02 μm^2^/s. However, instead of maintaining the low value, D_EGFR_^wt^ oscillated between 0.09 ± 0.02 and 0.23 ± 0.02 μm^2^/s. The oscillation displayed the periodicity of about 2.5 min and lasted for more than 20 min after the addition of EGF (the whole imaging duration). Since the diffusion coefficient of EGFR dimer was previously reported to be around half that of the monomer [14], we postulated that the periodical D_EGFR_ change reflects the monomer to dimer ratio changes. To directly determine the fractions of monomeric and dimeric eGFP-EGFR^wt^, we employed number and brightness (N&B) analysis [18]. Under resting conditions, the preformed dimer content was about 30% (Fig 1 panels C, D and E). After the addition of EGF, the monomer to dimer ratio was 0.5:0.5 at the time points which corresponded to the local D_EGFR_^wt^ minima (0.11 ± 0.03 μm^2^/s, t ≈ 1, 4, 6, 9, 12, 14, 17 min), whereas a ratio of 0.7:0.3 was observed at the local maxima (0.22 ± 0.03 μm^2^/s, t ≈ 2, 5, 7, 10, 13, 15, 20 min). Since the slow diffusing state (~0.11 μm^2^/s) always correlated with higher dimer content (50%) as opposed to the faster diffusing state (~0.22 μm^2^/s, ~30% dimer), we concluded that the D_EGFR_^wt^ oscillations reflected the monomer-dimer equilibrium shift, suggesting the presence of a negative feedback mechanism which monomerizes EGFR.

### Periodical change of monomer to dimer ratio occurs in the plasma membrane, independently from endocytosis

One of the feedback mechanisms that regulate EGFR signaling is endocytosis. It is detectable 2 to 5 min after the addition of EGF [19] thus occurs at a similar rate as the onset of D_EGFR_ oscillations. To evaluate the impact of endocytosis on D_EGFR_, we employed a C-terminally truncated mutant, EGFR^ΔC995^, which lacks several regions essential for endocytosis [20] including the AP-2 binding site L1010-L1011 [21], acetylation sites important for ubiquitin ligase binding (K1155, K1158, K1164) [22], and Grb2 binding sites (pY1068, pY1086) which can indirectly recruit E3 ubiquitin ligase Cbl [23]. As expected, eGFP-EGFR^ΔC995^ localized to the plasma membrane without being endocytosed upon the addition of EGF (S3 Fig panel A) although it still activated the ERK signaling pathway (S3 Fig panel B). On the other hand, RICS analysis revealed repetitive D_EGFR_^ΔC995^ changes that were similar to the D_EGFR_^wt^ patterns (S3 Fig panel C). We were able to conclude that EGFR monomerization took place on the plasma membrane, and was independent of endocytosis and receptor recycling.

Endosome formation was observed on the plasma membrane of cells expressing eGFP-EGFR^wt^ several minutes after the addition of EGF. Movement of endosomes was slow enough that it had been subtracted with the immobile fraction (S2 Movie). Accordingly, neither the confined diffusion of EGFR within endosomes, nor the movement of the endosomes themselves, contributed to the results presented here. Therefore, we exclusively analyzed the mobile EGFR fraction in the plasma membrane even if the endocytosis occurred during the experiment.

### Phospholipase Cγ participates in EGFR monomerization

**Fig 2:**
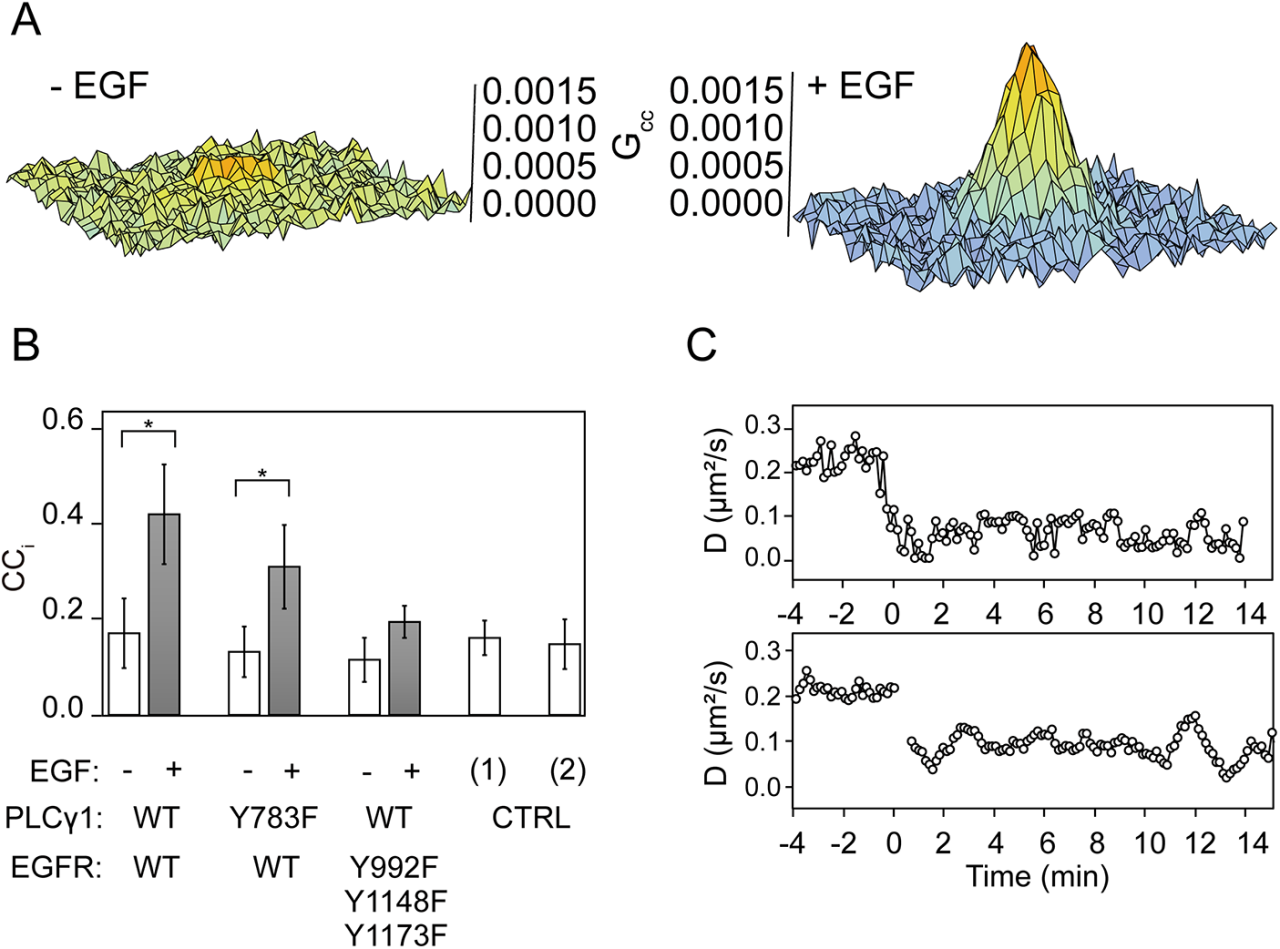
Participation of PLCγ1 in the feedback monomerization of the EGFR. (A) ccRICS analysis of PLCγ1 binding to EGFR upon EGF challenge. 2D cross-correlation of eGFP-EGFR^wt^ and mCherry-PLCγ1^wt^ in CHO-K1 cells before (left) and after (right) EGF addition. (B) Cross-correlation index (CC_i_) between mCherry-PLCγ1, eGFP-EGFR, and their mutants before (white) and after (grey) EGF addition. Lyn-mCherry + eGFP (1) and lyn-mCherry + lyn-eGFP (2) were used as negative controls (CTRL). Data are means ± SD. Symbol * indicates significant difference with p < 0.005 calculated using a two sided T-test with unequal variance. (C) Time traces of diffusion coefficient of eGFP-EGFR^Y992/1148/1173F^, lacking PLCγ binding sites in the upper panel, and eGFP-EGFR^wt^ coexpressed with mCherry-PLCγ1^Y783F^ (inactive mutant, lower panel).

Phospholipase Cγ (PLCγ) is recruited to the liganded EGFR [24] and plays a role in the EGFR signaling, as well as in its negative regulation [25]. To observe the interaction of PLCγ with EGFR, we applied cross-correlation RICS (ccRICS) [26] to CHO-K1 cells coexpressing eGFP-EGFR and mCherry-PLCγ1 (Fig 2A). Under resting conditions, mCherry-PLCγ1^wt^ was localized in the cytosol (S4 Fig panel A). The eGFP-EGFR/mCherry-PLCγ1^wt^ cross-correlation index [27] (*CC*_*i*_ = 0.18 ± 0.08) was indistinguishable from the negative controls (cytosolic eGFP and membrane targeted lyn-mCherry, *CC*_*i*_ = 0.17 ± 0.04; both membrane targeted lyn-eGFP and lyn-mCherry, *CC*_*i*_ = 0.15 ± 0.07), indicating no significant interaction of mCherry-PLCγ1 with eGFP-EGFR^wt^ (Fig 2B). Upon the addition of EGF, mCherry-PLCγ1^wt^ translocated to the plasma membrane and the *CC*_*i*_ substantially rose to 0.44 ± 0.11, indicating interaction of PLCγ with EGFR (Fig 2B, S4 Fig panel B).

To investigate the participation of PLCγ in EGFR monomerization, we made an EGFR mutant phosphodeficient at PLCγ binding sites (EGFR^Y992/1148/1173F^) [25, 28, 29]. The ccRICS between mCherry-PLCγ1 and coexpressed eGFP-EGFR^Y992/1148/1173F^ indicated no significant interaction under resting conditions (*CC*_*i*_ = 0.12 ± 0.05), or after addition of EGF (*CC*_*i*_ = 0.20 ± 0.04) (Fig 2B). These results together with the Ca^2+^ imaging data (S5 Fig) confirmed that EGFR^Y992/1148/1173F^ neither bound nor activated PLCγ1.

Under resting conditions, D_EGFR_^Y992/1148/1173F^ was similar to D_EGFR_^wt^ (0.22 ± 0.02 μm^2^/s). However, after the addition of EGF, D_EGFR_^Y992/1148/1173F^ dropped to 0.07 ± 0.02 μm^2^/s and remained low without any visible pattern (Fig 2C). Moreover, N&B analysis showed that approximately 50% of EGFR^Y992/1148/1173F^ was dimeric after EGF stimulation. To further evaluate the importance of pY992, pY1148 and pY1173, we employed a range of single and double phosphodeficient EGFR mutants (Table 1). Mutating a single PLCγ binding site had no detectable effect on D_EGFR_ under resting conditions or after the addition of EGF. Phosphodeficient mutations at two out of three tyrosine residues had slightly lower levels of EGRF monomerization, although the oscillatory pattern of D_EGFR_ was still visible in the presence of EGF. We concluded that phosphorylation of one out of three PLCγ binding sites was required and sufficient for the D_EGFR_ oscillations.

**Table 1:** Mutational study of the EGFR feedback monomerization at the PLCγ binding sites.

Construct	Mutation ^a^			Negative feedback^b^	EGF -	EGF +		
	Y992	Y1148	Y1173		D (μm2/s)	Dmin ^c^	Dmax ^d^	Davg ^e^
						(μm2/s)	(μm2/s)	(μm2/s)
EGFR^wt^				+ +	0.24 ± 0.02	0.11 ± 0.03	0.22 ± 0.03	0.17 ± 0.06
EGFR^Y992F^	F			+ +	0.24 ± 0.03	0.11 ± 0.03	0.21 ± 0.03	0.16 ± 0.05
EGFR^Y1148F^		F		+ +	0.24 ± 0.03	0.08 ± 0.04	0.21 ± 0.03	0.14 ± 0.07
EGFR^Y1173F^			F	+ +	0.24 ± 0.03	0.10 ± 0.04	0.22 ± 0.04	0.17 ± 0.07
EGFR^Y992/1148F^	F	F		+	0.22 ± 0.03	0.11 ± 0.02	0.19 ± 0.04	0.13 ± 0.05
EGFR^Y992/1173F^	F		F	+	0.24 ± 0.03	0.10 ± 0.02	0.19 ± 0.03	0.13 ± 0.04
EGFR^Y1148/1173F^		F	F	+ +	0.23 ± 0.03	0.11 ± 0.03	0.20 ± 0.03	0.15 ± 0.05
EGFR^Y992/1148/1173F^	F	F	F	-	0.22 ± 0.02	0.07 ± 0.02	ND ^f^	0.07 ± 0.02

^a^ F stands for the phosphodeficient mutation (phenylalanine substitution).

^b^ The strength of negative feedback scaled from as strong as for the EGFR^wt^ (+ +) to none (—).

^c, d^ Minimum (D_min_) and maximum (D_max_) level of the diffusion coefficient.

^e^ The diffusion coefficient averaged over the whole measurement time after EGF addition (D_avg_).

^f^ Not determinable.

Although the PLCγ binding residues of EGFR were crucial for the monomerization, these residues also interact with other signaling proteins [30–32]. To confirm the participation of PLCγ in EGFR monomerization, we used the EGFR-inactivatable PLCγ mutant, PLCγ1^Y783F^. This mutant lacks Y783 which is essential for EGFR-mediated PLCγ1 activation [33]. Under resting conditions, mCherry-PLCγ1^Y783F^ was mainly localized in the cytosol, and did not interact with eGFP-EGFR^wt^ (CC_i_ = 0.14 ± 0.06) (Fig 2B). Upon the addition of EGF, mCherry-PLCγ1^Y783F^ was recruited to the plasma membrane and interacted with eGFP-EGFR^wt^ (CC_i_ = 0.32 ± 0.09). This suggested that PLCγ1^Y783F^ most likely competed with endogenous PLCγ for binding to EGFR. As expected, the monomerization of EGFR was not observed in the cells coexpressing mCherry-PLCγ1^Y783F^, in which D_EGFR_ decreased from 0.24 ± 0.03 μm^2^/s to 0.07 ± 0.04 μm^2^/s upon the EGF addition and remained low, whereas the expression of mCherry-PLCγ1^wt^ had no influence on D_EGFR_ (Fig 2C, S4 Fig panel C).

### PKC counteracts EGFR dimerization

**Fig 3:**
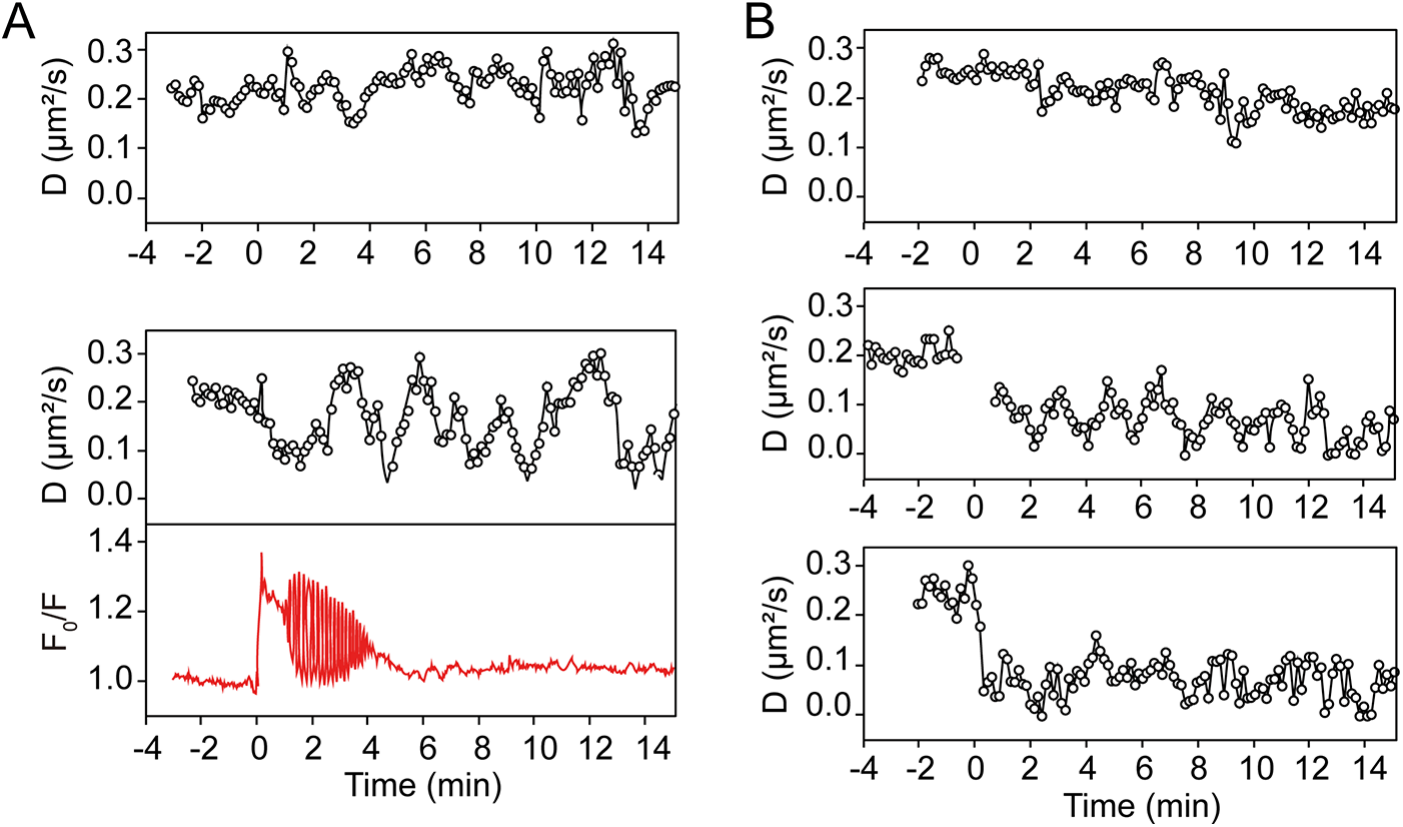
Participation of nPKC and PKD1 in the feedback monomerization of the EGFR. (A) Time traces of diffusion coefficient of EGFR^wt^ in the presence of the PKC activator PMA (upper) or under nominally Ca^2+^-free conditions (lower panel). In red, the time trace of cytosolic Ca^2+^ concentration monitored with Fura Red is shown. (B) Time traces of diffusion coefficient of eGFP-EGFR^wt^ in the cell coexpressing constitutively active mutant, mCherry-PKD1^S738/742E^ (upper), in the cell pre-treated with 10 μM CID755673, PKD1 inhibitor (middle), or in the cell coexpressing kinase deficient mutant, mCherry-PKD1^K612W^ (lower panel).

PLCγ hydrolyses phosphatidylinositol 4,5-bisphosphate into diacylglycerol (DAG) and inositol 1,4,5-trisphosphate (IP_3_). IP_3_ induces Ca^2+^ release from endoplasmic reticulum, whereas DAG along with Ca^2+^ recruit protein kinase C (PKC) to the plasma membrane and activates it [31, 34]. Additionally, PKC-mediated phosphorylation of T654 in the EGFR JM region downregulates signaling [35, 36]. To investigate the participation of PKC in the EGFR monomerization, we used the DAG mimetic PKC activator, phorbol 12-myristate 13-acetate (PMA). Pre-treatment with 200 nM PMA prevented EGFR dimerization upon the addition of EGF; D_EGFR_ before and after EGF addition was 0.25 ± 0.02 μm^2^/s and 0.23 ± 0.02 μm^2^/s, respectively (Fig 3A). This indicated that PKC activation perturbed EGFR dimerization.

PKCα is categorized as a classical PKC (cPKC) [37], and thus contains a C2 domain for Ca^2+^-dependent phospholipid binding. To investigate the participation of Ca^2+^ signaling in EGFR monomerization, we performed RICS analysis of eGFP-EGFR^wt^ expressing cells in nominally Ca^2+^-free Hank’s balanced salt solution (HBSS). In this solution, the cytosolic Ca^2+^ concentration increased upon the addition of EGF, but ceased in around 6 min due to the lack of store-operated Ca^2+^ entry (Fig 3A) [38], in contrast with a sustained or repetitive Ca^2+^ rise in standard HBSS (S2 Fig panel D). However, periodical changes of D_EGFR_ continued throughout the measuring period (>15 min) (Fig 3A). This indicated that the EGFR monomerization was Ca^2+^-independent providing an argument that most likely a novel PKC (nPKC) that lacks the C2 domain, rather than cPKC, contributed to the observed effect.

### Protein kinase D activation is essential for EGFR monomerization

Protein kinase D-1 (PKD1) is activated by nPKC-mediated phosphorylation of S738 and S742 in the activation loop [39–42]. Activated PKD1 then phosphorylates threonine residues at the JM region of EGFR, attenuating its signaling strength [43]. To examine the possible role of PKD1 in the monomerization of EGFR, we used PKD1^S738/742E^, a constitutively active phosphomimic mutant [40]. In the cells coexpressing eGFP-EGFR^wt^ and mCherry-PKD1^S738/742E^, around 70% of the EGFR was monomeric under resting conditions (D_EGFR_ = 0.26 ± 0.02 μm^2^/s), likewise in the cells expressing only EGFR^wt^. Upon the addition of EGF, however, no immediate dimerization was observed and D_EGFR_ had gradually decreased to 0.20 ± 0.03 μm^2^/s (Fig 3B).

To address the question whether lowered PKD1 activity influences EGFR monomerization, we used a PKD1 inhibitor, CID755673. At low concentration (≤10 μM), it has been previously demonstrated that CID755673 can specifically inhibit PKD1 in cultured cells without affecting the activity of other kinases including PKCα [44]. After 20 min of pre-incubation with 10 μM CID755673, cells expressing eGFP-EGFR^wt^ were subjected to the RICS measurement. CID755673 did not influence D_EGFR_ under resting conditions (0.21 ± 0.03 μm^2^/s). Upon addition of EGF, EGFR formed dimers without any monomerization occurring (D_EGFR_ = 0.07 ± 0.037 μm^2^/s, Fig 3B). Next, we employed a kinase-null PKD1 mutant (PKD1^K612W^) [45]. Overexpressed PKD1^K612W^ most likely competes with endogenous PKD for phosphorylation by nPKC. In cells coexpressing PKD1^K612W^, D_EGFR_ decreased from 0.25 ± 0.03 μm^2^/s to 0.08 ± 0.04 μm^2^/s upon the addition of EGF, and no monomerization was observed (Fig 3B). The fraction of dimers in the presence of EGF was no less than 60% 4 min after EGF addition, which is slightly higher than at the local D_EGFR_^wt^ minimum without PKD1^K612W^ coexpression. These results indicate that PKD1 is a key molecule for the monomerization of EGFR.

### EGFR juxtamembrane domain phosphorylation is crucial for monomerization

**Fig 4:**
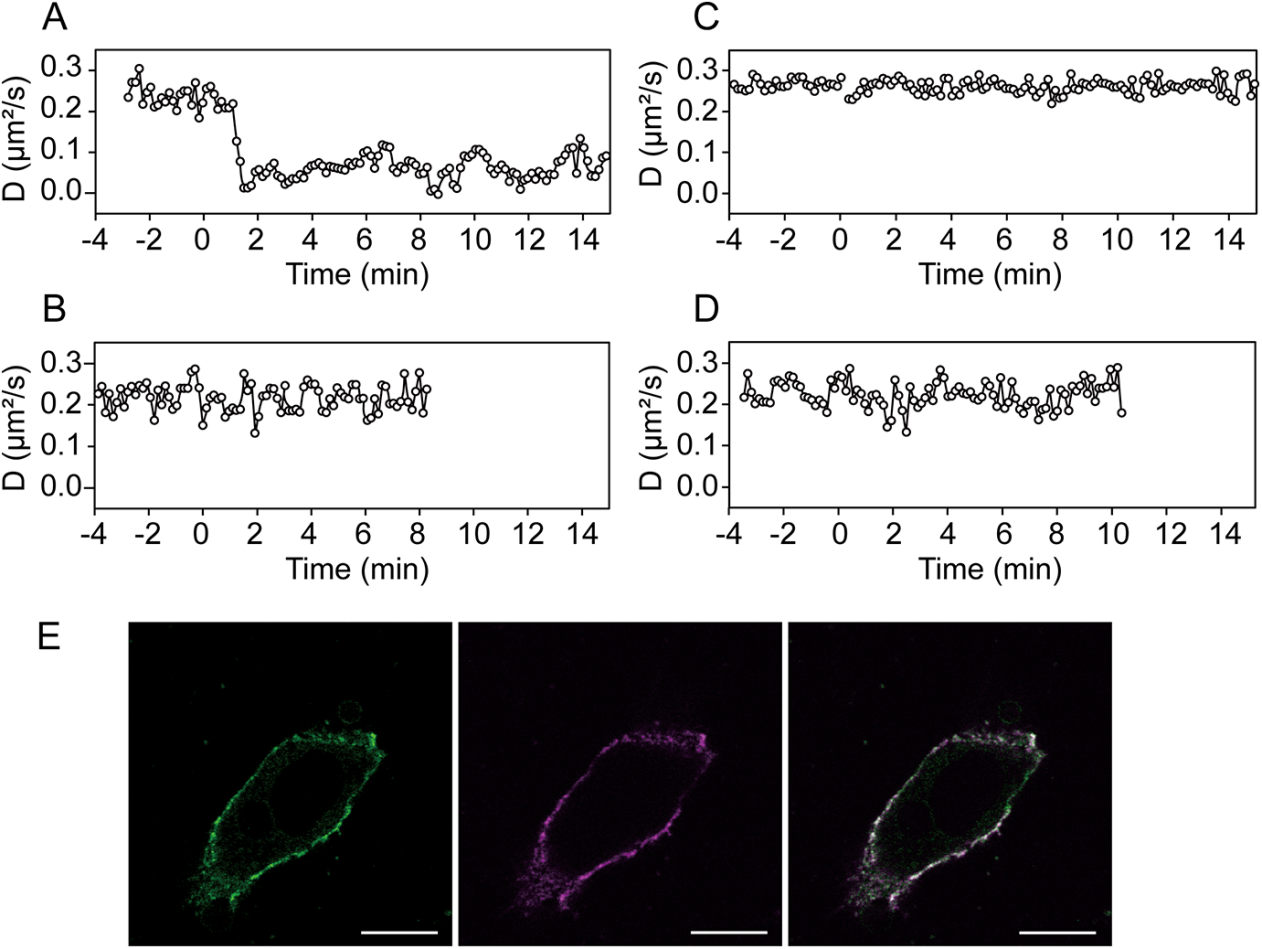
Threonine phosphorylation at JM regions influences the monomer–dimer equilibrium of EGF-bound EGFR. (A-D) Time traces of the diffusion coefficient of the EGFR with JM region threonine residue mutations; phosphodeficient mutant, EGFR^T654/669A^ (A), three phosphomimic mutants, EGFR^T654/669E^ (B), EGFR^T654E^ (C), and EGFR^T669E^ (D). (E) EGF (Alexa647-labeled, 0.17 μM; magenta) binding to EGFR^T654/669E^ (green) expressing cell. The right panel shows the merged image. Scale bars represent 10 μm.

PKD1 phosphorylation sites in the EGFR juxtamembrane segment, T654 and T669 [43], are located in the JM-A and JM-B regions, respectively. We performed mutational analysis of these two threonine residues (Table 2). EGFR phosphodeficient at both T654 and T669 (EGFR^T654/669A^) formed dimers but did not monomerize, indicating that phosphorylation of T654 and/or T669 is essential for the monomerization (Fig 4A). Both phosphodeficient mutants with a single mutation at either EGFR^T654A^ or EGFR^T669A^ retained the oscillatory monomerization. Phosphomimic glutamate substitution of either T654 (EGFR^T654E^) or T669 (EGFR^T669E^) caused EGFR to stay in a monomeric state after EGF addition (Fig 4C and 4D). This indicated that phosphorylation of one of these threonine residues is an essential step in the EGFR feedback monomerization. Even a single phosphorylation event appeared sufficient to shift the monomer-dimer equilibrium towards monomeric form (Table 2). This was not due to negative regulation of EGF binding, since the double phosphomimic mutants (EGFR^T654/669E^) preserved affinity to EGF despite staying in monomeric state (Fig 4B and 4E).

**Table 2:** Mutational study of the EGFR monomer-dimer equilibrium at the JM domain phosphorylation sites.

Construct	Mutation ^a^		Monomer-dimer equilibrium shift^b^	EGF -	EGF +	
	T654	T669		D	D_min_ ^c^	D_max_ ^d^
				(μm^2^/s)	(μm^2^/s)	(μm^2^/s)
EGFR^wt^			oscillation	0.24 ± 0.02	0.11 ± 0.03	0.22 ± 0.03
EGFR^T654A^	A		oscillation	0.25 ± 0.03	0.11 ± 0.02	0.25 ± 0.02
EGFR^T654E^	E		monomer	0.27 ± 0.02	ND ^e^	0.26 ± 0.03
EGFR^T669A^		A	oscillates	0.25 ± 0.02	0.09 ± 0.05	0.24 ± 0.02
EGFR^T669E^		E	monomer	0.24 ± 0.03	ND	0.23 ± 0.03
EGFR^T654/669A^	A	A	dimer	0.24 ± 0.02	0.09 ± 0.04	ND
EGFR^T654/669E^	E	E	monomer	0.24 ± 0.03	ND	0.23 ± 0.03

^a^ A and E stand for the phosphodeficient and phosphomimic mutation (alanine and glutamate substitution, respectively).

^b^ The state towards which the monomer-dimer equilibrium is shifted after EGF addition.

^c, d^ Minimum (D_min_) and maximum (D_max_) level of the diffusion coefficient.

^e^ Not determinable.

### ERK activity is attenuated by EGFR feedback monomerization

**Fig 5:**
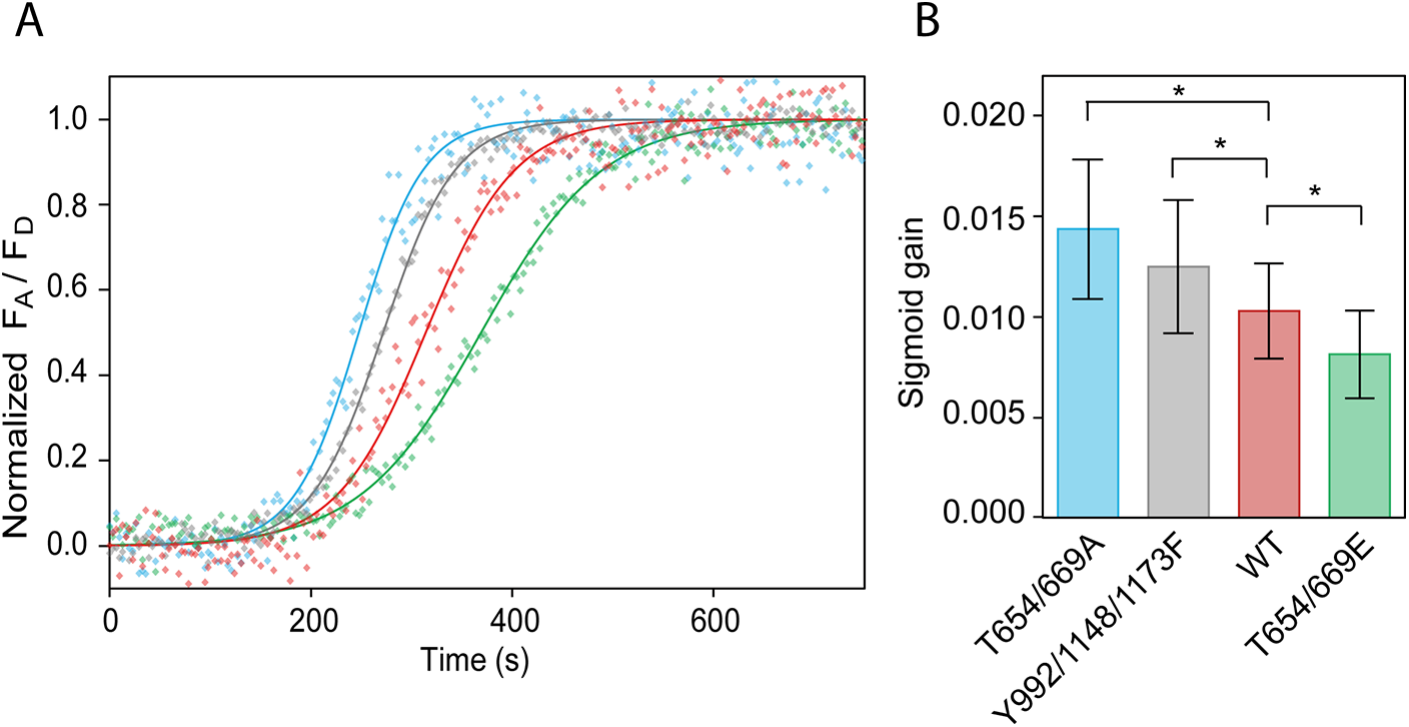
ERK activity correlates with the level of EGFR dimerization. ERK activity was monitored with EKAREV. (A) Time traces of the normalized fluorescence intensity ratio of the acceptor over the donor channel (dots) fitted to the sigmoid function (lines). Sample curves, showing the median values of sigmoid gain for EGFR^T654/669A^ (blue), EGFR^Y992/1148/1173F^ (grey), EGFR^wt^ (red), and EGFR^T654/669E^ (green) are shown. (B) Sigmoid gains as indices of the ERK activity. The data are means ± SD. The symbol * indicates significant differences with p < 0.005 calculated using a two sided T-test with unequal variance.

The Ras-Raf-MEK-ERK signaling cascade couples signals from plasma membrane receptors, such as EGFR, to transcription factors that regulate gene expression. To evaluate whether EGFR monomerization affects the signaling strength, we compared the ERK activity in CHO-K1 cells expressing eGFP-EGFR^wt^, eGFP-EGFR^Y992/1148/1173F^, eGFP-EGFR^T654/669A^ and eGFP-EGFR^T654/669E^. ERK activity was monitored using Förster resonance energy transfer (FRET) based indicator EKAREV [46] and analyzed by fitting the time traces of FRET efficiency to the sigmoid function. The sigmoid gain (*α*) was used as an ERK activity index. Compared to EGFR^wt^ [*α* = (1.0 ± 0.3) × 10^−2^] (Fig 5), cells expressing the phosphodeficient mutant EGFR^T654/669A^ showed higher ERK activity [*α* = (1.4 ± 0.3) × 10^−2^, p < 0.005] whereas expression of the phosphomimic mutant, EGFR^T654/669E^, resulted in lower activity [*α* = (0.8 ± 0.2) × 10^−2^, p < 0.005]. Furthermore, cells expressing EGFR^Y992/1148/1173F^, which shows no EGFR monomerization, also showed higher ERK activity than EGFR^wt^ [*α* = (1.2 ± 0.2) × 10^−2^, p < 0.005]. We concluded that the monomer to dimer ratio correlated with the EGFR signaling strength monitored via ERK activity, thus feedback monomerization downregulated ERK activity.

## Discussion

**Fig 6:**
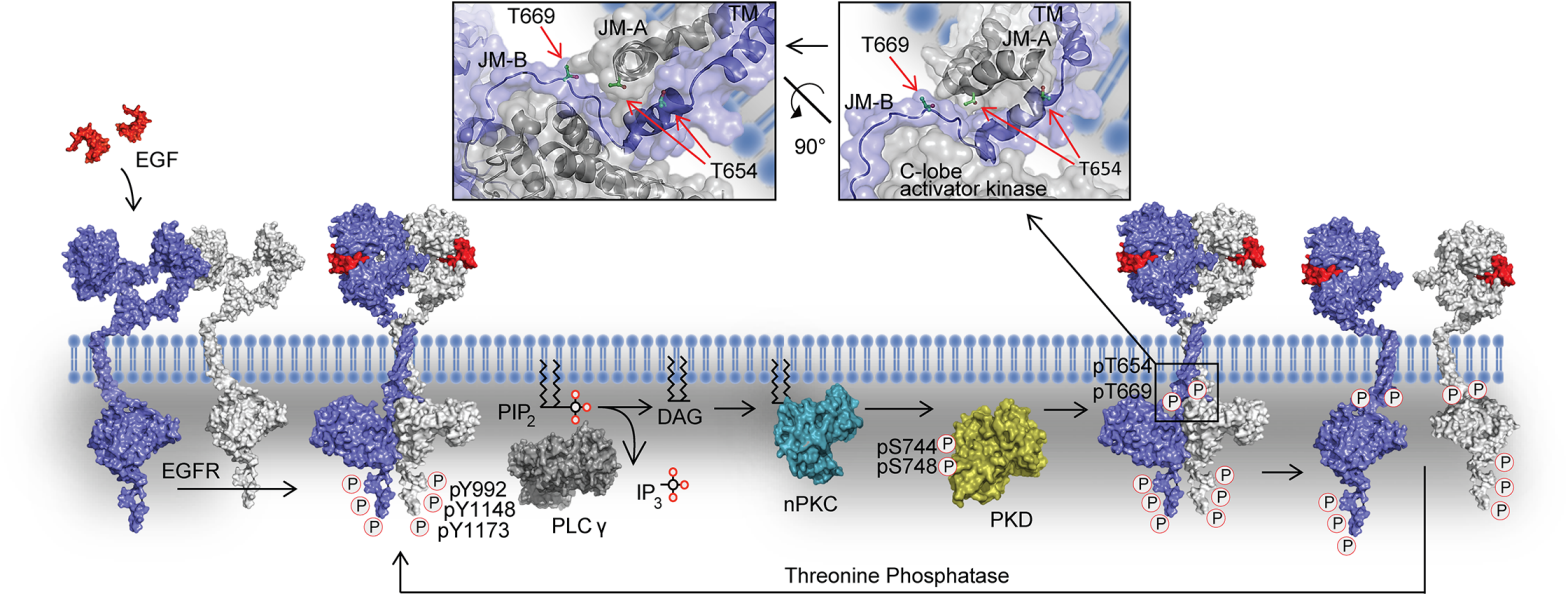
Proposed mechanism of EGFR feedback monomerization upon EGF stimulation. After EGF challenging, two EGFR monomers associate on the plasma membrane to form an asymmetric dimer. The asymmetric dimer formation activates the kinase domain which transphosphorylates tyrosine residues at the C-termini of the receptor, three of which (pY1173, pY1148, pY992) recruit PLCγ1. EGFR phosphorylates PLCγ1 on Y783 for activation. PLCγ1 hydrolyses PIP_2_ forming DAG. DAG activates nPKCs, which phosphorylates PKD at S744 and S748 for activation. PKD causes EGFR phosphorylation at T654 and/or T669 to shift the monomer-dimer equilibrium of liganded EGFR back towards the monomer. The insets show the close-up of the JM part. The two PDB files (2M20, 3GOP) were superimposed and aligned in the JM-A region to obtain presented images.

By introducing a time-lapse approach to RICS and applying it to study EGFR, we continuously monitored the diffusion coefficient of the transmembrane proteins with a temporal resolution high enough to monitor processes happening in the order of minutes. This analysis can be performed over a time period long enough to trace signal progression after the addition of EGF. Using CHO cells as a model system, we demonstrated that EGFR displayed periodical changes of diffusion coefficient in the presence of EGF. N&B analysis showed that this oscillatory change of D_EGFR_ reflected the shift in the monomer-dimer equilibrium. The equilibrium was regulated through the phosphorylation of T654 and T669 of EGFR via the PLCγ1-nPKC-PKD pathway. We propose a novel negative feedback mechanism whereby ligand bound EGFR is monomerized to control the signaling output (Fig 6).

SPT [47] and FRAP [48] studies have shown that EGFR can undergo fast diffusion, slow diffusion or stay immobile. In this study, D_EGFR_ under resting conditions was determined to be about 0.24 μm^2^/s by RICS, which falls in the fast diffusion range. Both the slow and immobile diffusion modes were eliminated from the RICS analysis through the moving average subtraction, but the majority of EGFR (about 80%) was found in the mobile fraction and thus subjected to analysis. Due to very low D_EGFR_ values, we encountered difficulties in deconvolution of the two components by RICS. We considered the determined D_EGFR_ as the weighted average of both components and attributed the observed changes to the shift in monomer-dimer equilibrium, what was directly confirmed by N&B analysis of the same data set. The N&B analysis is based on the fluctuation of fluorescence intensity in respective pixels while being independent of the diffusion coefficient. By using N&B analysis, Nagy et al. have reported that as much as 30% of EGFR is present as preformed dimer in CHO cells expressing >5 ×10^5^ receptors whereas EGFR is monomeric under resting conditions for the expression levels of 0.5–2 ×10^5^ receptors per cell [49, 50]. In our experimental setup, although the expression level was relatively low (0.7–2.5 ×10^5^), the dimer fraction was 0.3 under resting conditions. The apparent discrepancy might be caused by different experimental conditions such as a position of labeling with fluorescent protein (C- or N-terminus). N&B analysis also revealed that upon the EGF stimulation, the dimer fraction increased to 0.5. Intriguingly, the minimum value of the diffusion coefficient during the oscillation (ca. 0.09 μm^2^/s) was less than half of the maximum value (ca. 0.24 μm^2^/s), although the fluctuation of the dimer fraction is only between 0.5 (minimum diffusion coefficient) and 0.3 (maximum diffusion coefficient). This implies greater difference between the diffusion coefficients of monomer and dimer than the values estimated from the radius of the membrane-crossing domain [13]. Low-Nam et al. have reported marked slow down of ligand-bound EGFR dimers as compared to ligand bound dimers whose catalytic activity is inhibited, and concluded that reduced mobility is a complex reflection of the stability and size of the protein aggregate as well as signaling-mediated changes in the local environment [51]. The oscillation of diffusion coefficient observed in this study might also reflect change in the state of the protein aggregation and the local environment.

Mutational analysis of the EGFR C-terminus pointed to three phosphorylatable tyrosine residues, Y992, Y1148, Y1173, that are responsible for the negative feedback monomerization. These residues are known to be PLCγ1 binding sites, but also as docking sites for tyrosine protein phosphatase non-receptor type 1 (PTP-1B; pY992, pY1148) [52], signal transducer and activator of transcription 5 (Stat-5; pY992) [53], SH2 domain containing transforming protein (Shc; pY1148, pY1173) [54], SH2 domain containing protein tyrosine phosphatase 1 (SHP-1; pY1173) [55], and downstream of kinase-related protein (Dok-R; pY1148) [56]. Among them, PTP-1B interacts with EGFR exclusively on the endoplasmic reticulum [52], and hence cannot influence the EGFR monomerization on the plasma membrane. Analyses of double phosphodeficient mutants (Table 1) proved that the availability of even one out of three phosphorylation sites is sufficient for the feedback monomerization to occur. We concluded that PLCγ1, the only protein known to bind to all three tyrosine residues, took part in EGFR monomerization. Furthermore, we also confirmed the participation of the PLCγ1 downstream proteins (nPKC and PKD).

Since EGFR monomerization was Ca^2+^-independent and at the same time responsive to PMA, a DAG-mimicking PKC activator, we hypothesized that nPKC plays an important role in the investigated mechanism. To further examine this hypothesis, we tested the influence of PKD which is activated by nPKC [39–42] and phosphorylates EGFR [43]. PKD has been classified as a Ca^2+^/calmodulin-dependent protein kinase (CAMK) due to its sequence homology with *Dictyostelium* myosin light-chain kinase [57, 58]. However, none of the reported activation mechanisms are directly Ca^2+^-dependent, and Ca^2+^ itself is not considered to control PKD activation since PKD does not bind it [59]. At the plasma membrane, DAG production leads to PKD recruitment and activation. Nonetheless DAG alone is not sufficient for PKD activation, since phosphorylation of its activation loop, at S744 and S748, is necessary to induce kinase activity. This phosphorylation is catalyzed via upstream kinases such as nPKC [59].

We revealed that two threonine residues, T654 and T669, in the JM segment of EGFR were crucial for feedback monomerization. PKCα- and ERK-mediated phosphorylation of T654 and T669, respectively, are known to downregulate the EGFR tyrosine kinase activity [36, 60, 61]. Moreover, stimulation with platelet-derived growth factor causes PKD to phosphorylate EGFR at T654 and T669, which leads to suppression of EGF-mediated c-Jun N-terminal protein kinase activation [43]. The phosphorylation of T654 by PKC provides an inhibition mechanism of EGFR transactivation by G-protein coupled receptor agonist [62]. However, the precise mechanisms that downregulate the EGFR pathway via T654/669 phosphorylation have been obscure. We show that the phosphorylation of T654/669 shifts the monomer-dimer equilibrium of ligand bound EGFR towards monomers, which downregulates the strength of the signal without loss of affinity to EGF.

In this study we revealed the negative feedback pathway (Fig 6) that inhibits EGFR dimerization by threonine phosphorylation. This cascade of events provides the necessary time delay between EGFR dimerization, activation, and monomerization, inactivation, which gives rise to the oscillatory behavior in the observed time scale. However, the recovery process that allows for new EGFR dimerization remains to be revealed to complete the feedback loop. Most probably pT654/669 dephosphorylation is crucial in this process. Nonetheless, to the best of our knowledge, no protein phosphatase that dephosphorylates EGFR T654/669 has been reported so far. Protein phosphatase 2A (PP2A) could be responsible for the recovery phase of the feedback monomerization loop, as it is abundantly expressed in eukaryotic cells [63] and interacts with EGFR [64]. Additionally, dephosphorylation of the C-terminal tyrosine residues is required to suppress downstream signals. In general, the balance between kinase and phosphatase activities determines the level of tyrosine phosphorylation. Since feedback monomerization turns off the kinase activity, the phosphorylation level of EGFR should be lowered without regulating the phosphatase activity. A number of protein tyrosine phosphatase (PTPs) are reported to dephosphorylate EGFR, some of which (RPTPσ, RPTPκ, LAR, DEP-1, LRP) are anchored to the plasma membrane [65, 66]. Taking into account the substrate specificity of PTPs [67] and numerous tyrosine phosphorylation sites of EGFR, multiple PTPs are possibly responsible for C-terminus dephosphorylation.

The molecular mechanisms causing EGFR monomerization by means of T654/T669 phosphorylation are not yet fully understood. T654 is located in the JM-A segment, and is involved in the formation of the antiparallel helical dimer (Fig 6). In the JM-A segment, ^655^LRRLL^659^ consolidates the antiparallel helices, where hydrophobic interactions between L655 and L658 stabilize the structure [9]. On the surface of the antiparallel helices, the positively charged R656 side chain contributes to the interaction with the negatively charged plasma membrane. The addition of a negatively charged phosphate group to T654 might weaken an interaction between the antiparallel helices and lipid bilayer. It might also disturb the JM-A helical structure. T669 is in the JM-B segment, and is involved in forming the latch around the C-lobe of the activator kinase domain (Fig 6). Phosphorylation of T669 changes the charge, and thus the conformation, of the latch, which may weaken the interaction between the JM-B and the activator kinase domain. The quaternary structure at the latch domain is critical to stabilize the dimer; it has been reported that alanine substitution of R953, in the region of activator kinase interacting with the JM-B of the receiver, abolishes latch docking [9]. In this article, we are reporting the monomerization of the EGFR upon the threonine phosphorylation. Also, it has been recently described that inhibition of tyrosine phosphorylation, either by tyrosine kinase inhibitors or by overexpression of tyrosine phosphatase, stabilizes the EGFR dimer [68]. Thus, phosphorylation states of EGFR might play a role as a key regulator of the monomer-dimer equilibrium of EGFR. To fully understand the mechanism of the EGFR monomerization, information of dynamic structure of the juxtamembrane regions is necessary. As the experimental acquisition of the dynamic structure data is difficult, atomistic molecular dynamics simulation [69] might be applicable for predicting the dynamic structure.

In this study, we confirmed the PLCγ-nPKC-PKD1 pathway as a negative feedback loop to phosphorylate EGFR at T654/T669. Although T654 is also a PKCα phosphorylation site, we concluded that PKCα did not play a significant role in the EGFR feedback monomerization in our experimental system for the following reasons: (i) monomerization was Ca^2+^-independent, (ii) complete arrest of EGFR monomerization was achieved using the PKD inhibitor, CID755673, at the concentration which selectively blocks PKD activity, (iii) coexpression of the kinase-null PKD1^K612W^ blocked EGFR^wt^ monomerization. These findings by no means exclude PKCα-mediated T654 phosphorylation from attenuating the EGFR signal in other types of cells expressing a different repertoire of proteins.

Feedback pathways are crucial for precise signal regulation. Immediate regulatory mechanisms engage preexisting components, whereas later stage mechanisms rely on transcriptional response [19, 70, 71]. Clathrin-mediated endocytosis (CME) is an immediate negative feedback pathway for EGFR stimulated by a low concentration agonist mimicking physiological conditions. Endosome incorporated EGFRs are either trafficked to a lysosome for degradation or recycled back to the plasma membrane (t_1/2_ = 10–23 min) [72]. The EGFR monomerization found in this study could underlie an additional immediate negative feedback mechanism. The onset of the feedback monomerization is in the order of minutes, thus in the same time range as CME. On the other hand, the whole process of the monomerization pathway takes place on the plasma membrane with a periodicity of around 2.5 min, which is up to ten times faster than the EGFR recycling rate from the endosome. Moreover, ligand-free EGFR monomers can also be phosphorylated at T654/T669, in contrast to CME where the EGFR molecules auto-phosphorylated at the C-terminus are sorted to the endosomes. The receptor monomerization by threonine phosphorylation observed in this study reflects a novel, fast negative feedback mechanism that regulates EGFR activity. This mechanism is expected to participate in an orchestrated regulation of the EGFR activity along with CME and other regulatory pathways to fine-tune the signaling level.

## Materials and Methods

### Plasmid construction

Refer to S1 Text for plasmid construction.

### Cell culture and protein expression

All experiments were performed using Chinese Hamster ovary cells, CHO-K1 (Cat. No 85051005; European Collection of Cell Cultures, Salisbury, UK), which do not express endogenous EGFR. Cells were cultured in Ham's F-12 Nutrient Mixture (Sigma-Aldrich, St. Louis, MO) supplemented with 10% fetal bovine serum (Life Technologies, Carlsbad, CA) and 50 μg/ml gentamycin (Life Technologies) at 37°C under 5% CO_2_ atmosphere. Two days prior to imaging, cells were seeded on a glass bottom 8-well Lab-Tek chamber slide (Thermo Scientific, Rockford, IL) and transfected with one or combination of following plasmids: eGFP/pcDNA3, Lyn-eGFP/pcDNA3, Lyn-mCherry/pcDNA3, wild type or mutant eGFP-EGFR/pcDNA3, mCherry-HA-PDK1/pcDNA3, PLCγ1-mCherry/pcDNA3, pEKAREV [46] (~0.2 μg of each plasmid per well) using FuGENE6 transfection reagent (Promega, Madison, WI) according to the manufacturer’s protocol. The EGFR expression level was evaluated by the confocal microscopy. Before imaging, cells were incubated in serum-free F-12 for ≥ 20 min for serum starvation. For PKC activation experiments, cells were incubated for ≥ 20 min in serum-free F-12 containing of 200 nM phorbol 12-myristate 13-acetate (PMA; InvivoGen, Toulouse, France) and 0.02% Kolliphor EL (Sigma-Aldrich). For PKD inhibition experiments, cells were incubated with 10 μM CID755673 (Sigma-Aldrich) in the presence of 0.02% Kolliphor EL.

### Confocal image acquisition

All the confocal images were acquired with a laser scanning microscope (Fluoview FV1000; Olympus, Tokyo, Japan) using a water immersion objective lens, UPLSAPO 60X W NA = 1.20. Proteins of interest (EGFR, PKD1, PLCγ1) were labeled with eGFP or mCherry for imaging purposes. A 488-nm laser line emitted from an argon laser and a 559-nm diode-pumped solid-state laser was used to excite eGFP and mCherry, respectively. The excitation beam was reflected by a main dichroic mirror (DM405/488/559/635). The emission was split into eGFP and mCherry channels by using a second dichroic mirror (SDM560) and detected through a band-pass filter BA505-540 for eGFP and BA575-675 for mCherry.

### Ca^2+^ imaging

A Ca^2+^ indicator, Fura Red, was loaded into the cells expressing eGFP-EGFR by the incubation of cells for 30 min in HBSS (1.26 mM CaCl_2_, 0.49 mM MgCl_2_, without phenol red; Life Technologies) containing 10 μM Fura Red AM (Life Technologies) and 0.02% Kolliphor EL. The imaging was performed with the Fluoview FV1000 using either UPLSAPO 60X W NA = 1.20 objective lens (when Ca^2+^ imaging was performed along with raster image acquisition) or UPLSAPO 20X NA:0.75 (in the case of individual Ca^2+^ imaging experiment). Both Fura Red and eGFP were excited using the 488-nm laser line. The main dichroic mirror used was DM405/488. The emission was split into eGFP and Fura Red channels using a dichroic mirror SDM560 and detected through a band-pass filter BA505-540 for eGFP and BA575-675 for Fura Red.

### Observation of EGF binding

CHO-K1 cells expressing eGFP-EGFR^wt^ or eGFP-EGFR^T654/669E^ were exposed to 0.17 μM Alexa Fluor 647 conjugated EGF (Life Technologies) and immediately fixed with 4% ice-cold formaldehyde (Thermo Scientific). Imaging was performed with the Fluoview FV1000 using UPLSAPO 60X W NA = 1.20 objective lens. The 488-nm laser line and a 635-nm diode laser was used to excite eGFP and Alexa Fluor 647, respectively. The excitation beam was reflected by a main dichroic mirror (DM405/488/559/635). The emission was split into two channels by using a second dichroic mirror (SDM560) and detected through a BA505-540 band-pass filter for eGFP and BA655-755 for Alexa Fluor 647.

### Functional imaging of extracellular signal-regulated kinase activity

To monitor the ERK activity, we used a FRET probe, EKAREV [46] (gift from Dr. Matsuda). Time-lapse images were acquired with the Fluoview FV1000 using UPLSAPO 60X W NA = 1.20. The EKAREV probe was excited with a 440-nm laser diode. The main dichroic mirror (DM405-458/515/559/635) was used to reflect the excitation beam. The emission was split into the donor (eCFP) and the acceptor (Ypet) channels using SDM510 dichroic mirror, and detected through a BA455-500 or BA505-605 band-pass filters for donor or acceptor channels, respectively. The fluorescence intensity ratio of the acceptor (Ypet) over the donor (eCFP) channel, reflecting the FRET efficiency, was calculated. In one experiment, we simultaneously monitored up to 70 cells. The time traces of the fluorescence intensity ratio were normalized between 0, which represented the state before EGF stimulation and 1, which was the level of probe saturation. The normalized time traces were fitted to a sigmoid function using OriginPro 8 (OriginLab, Northampton, MA):

$$y=\frac{1}{1+10^{-\left(t-t_{0.5}\right)\alpha }}$$ (1)

where *t* is the time; *t*_*0*.*5*_ is the 50% saturation time; and *α* is the sigmoid gain, further used to evaluate ERK activity.

### Raster image correlation spectroscopy

Immediately before installing a sample on the microscope, the growth medium was replaced with HBSS. Raster images of the basal cell membrane were acquired with the Fluoview FV1000 operated in the photon counting mode (using the same optical settings as for the confocal imaging). The sample was continuously scanned (256 × 256 of 50 nm pixels) for approximately 30 min using 20 μs pixel dwell time. At around 5 min, EGF was added to the final concentration of 0.17 μM. All the images were acquired at controlled room temperature (≈ 25°C).

Fifty sequential images were used as a time bin for calculating RICS auto-correlation with SimFCS [73]. Overlapping time bins with an interval of 5 frames between the first frames of each bin were subjected for the time-series analysis. In order to compensate for slow cell movement and to filter out the immobile fraction, 10 frames of moving average were subtracted from the respective images. Beneficial for signal to noise ratio was averaging the intensity among a small (5–10) number of frames. The auto-correlation calculated for each time bin was fitted to the single component 2D model of membrane-bound diffusion (S1 Text) to determine the diffusion coefficient, *D*.

We determined the cross-correlation amplitude, *G(0*,*0)*_*cc*_, and auto-correlation amplitudes for the green and red channels [*G(0*,*0)*_*green*_ and *G(0*,*0)*_*red*_, respectively] from 100 consecutive raster images. To quantify the interaction between eGFP and mCherry labeled proteins, the cross-correlation index (*cc*_*i*_) [26] was calculated as follows:

$$cc_{i}=\frac{G{\left(0,0\right)}_{cc}}{\frac{1}{2}\times \left(G{\left(0,0\right)}_{green}+G{\left(0,0\right)}_{red}\right)}$$ (2)

To estimate the background cross-correlation index, we performed a series of the negative control experiments: (i) combination of cytosolic eGFP and membrane-targeted mCherry (eGFP/lyn-mCherry) which is the same configuration as EGFR-PLCγ interaction, and (ii) combination of membrane-targeted eGFP and mCherry (lyn-eGFP/lyn-mCherry) for non-interacting membrane proteins. For details, refer to S1 Text.

### Number and brightness analysis

To analyze the monomer/dimer state of the receptor, the stacks of images acquired for RICS were subjected for the N&B analysis [18] which uses the apparent molecular brightness (*B*)–the ratio of the fluorescence signal variance (*σ* ^2^) to its average intensity (⟨*k*⟩) in the respective pixel over many frames. *B* is directly related to the true molecular brightness (ε, counts/pixel dwell time/molecule) of fluorescent molecules occupying the pixel (in N&B both EGFR monomer and dimer are regarded as single molecules) as follows:

$$B=\frac{\sigma^{2}}{\left\langle k\right\rangle }=\varepsilon +1$$ (3)

A histogram of *B* versus ⟨*k*⟩ calculated with SimFCS to estimate the monomer to dimer ratio [18, 74]. We assumed that monomeric eGFP-EGFR shows *ε* equal to that of monomeric eGFP which increases 2-fold for eGFP-EGFR dimer. *ε* of monomer eGFP (≈ 0.1 counts/dwell time/molecule) was determined using monomeric eGFP in solution and in the cytoplasm. For details, refer to S1 Text.

### Evaluation of Y1173 phosphorylation by immunostaining

CHO-K1 cells expressing eGFP-EGFR^wt^, were incubated in serum-free F-12 for 20 min for serum starvation and subsequently treated with 0.17 μM EGF (Sigma-Aldrich) for 5 min then fixed with 4% ice-cold formaldehyde. After permeabilization with 0.1% Triton X-100 (Sigma-Aldrich), and blocking with 0.2% BSA (Life Technologies), cells were stained with rabbit anti-human phospho-EGFR pY1173 primary antibody (BioCat, Heidelberg, Germany) and then with goat anti-rabbit IgG (H+L) secondary antibody, Alexa Fluor 647 conjugate (Life Technologies).

Imaging was performed with the Fluoview FV1000 using UPLSAPO 60X W NA = 1.20 objective lens. The 488-nm laser line and a 635-nm diode laser was used to excite eGFP and Alexa Fluor 647, respectively. The excitation beam was reflected by a main dichroic mirror (DM405/488/559/635). The emission was split into two channels by using a second dichroic mirror (SDM560) and detected through a BA505-540 band-pass filter for eGFP and BA655-755 for Alexa Fluor 647.

For evaluation of Y1173 phosphorylation in the presence or absence of EGF, we used the fluorescence intensity of eGFP and Alexa Fluor 647 (S2 Fig panel B).

## Supporting Information

S1 Fig Determination of the expression level in respective cells from fluorescence intensity. (A) Horizontal profiles of RICS autocorrelation amplitudes for eGFP solutions with different concentrations. Number of the molecules in the confocal volume (N) calculated using G(0,0) for respective curves is presented on the right. (B) The calibration curve showing the relationship between N and the fluorescence intensity.
(TIF)

S2 Fig Evaluation of eGFP-EGFR functionality. (A) EGF (Alexa647-labeled, 0.17 μM; magenta) binding to eGFP-EGFR^wt^ (green) expressing cells but not to the control, non-transfected, cells seen on differential interference contrast image (DIC). Right panel shows both channels merged with a DIC image. Scale bars equal to 20 μm. (B) Phosphorylation of eGFP-EGFR at Y1173. Cells expressing eGFP-EGFR^wt^ were immunostained using rabbit anti-human phospho-EGFR pY1173 antibody and Alexa647-labeled goat anti-rabbit IgG antibody. The plot shows fluorescence intensity of Alexa 647 versus eGFP for quiescent (blue) and EGF challenged (orange) cells. The slope of F_Alexa 647_ over F_eGFP_ is presented in the inset (error bars indicate SEM). (C) Time trace of average fluorescence intensity of the eGFP-EGFR^wt^ on the plasma membrane, at t = 0, EGF was added. (D) Cytosolic Ca^2+^ concentration monitored with Fura Red. Reproducible Ca^2+^ response in cells expressing eGFP-EGFR^wt^ (black) and non-transfected control cells (red). (E) ERK activity monitored with EKAREV in CHO-K1 cells expressing eGFP-EGFR^wt^ (black) and control cells (no eGFP-EGFR^wt^ expression, red). We analyzed 53 and 66 cells (for eGFP-EGFR^wt^ expressing and control cells, respectively) with essentially the same results.
(TIF)

S3 Fig EGFR feedback monomerization is not related to the endocytosis and takes place on the plasma membrane. (A) Confocal images of the Z-projection and cross sections of eGFP-EGFR^ΔC995^ in CHO-K1 cells before (left) and 30 min after EGF addition (right). After 30 min addition of EGF, eGFP-EGFR^ΔC995^ was still exclusively targeted to the plasma membrane and no endosomes were observed. Scale bars indicate 10 μm. (B) ERK activity monitored with EKAREV in CHO-K1 cells expressing eGFP-EGFR^ΔC995^ (black) and non-transfected control cells (red). (C) Reproducible time trace of eGFP-EGFR^ΔC995^ diffusion coefficient. EGF (0.17 μM) was added at the t = 0.
(TIF)

S4 Fig Coexpression of mCherry-PLCγ1^wt^ does not influence the diffusion coefficient of eGFP-EGFR^wt^ in CHO-K1 cells. (A, B) Confocal images of the cells coexpressing eGFP-EGFR^wt^ (green) and mCherry-PLCγ1^wt^ (red) before (A) and immediately after EGF addition (B). Upon EGF challenging, the recruitment of mCherry-PLCγ1^wt^ to the plasma membrane was observed, most probably through the binding to eGFP-EGFR^wt^. (C) The diffusion coefficient of eGFP-EGFR^wt^ in the cell coexpressing mCherry-PLCγ1^wt^.
(TIF)

S5 Fig Ca^2+^ response in cells expressing eGFP-EGFR^Y992/1148/1173F^ monitored with Fura Red. No Ca^2+^ response was observed in 31 out of 39 cells (upper left). Weak continuous response was observed in 1 cell (upper right), immediate oscillations with low amplitude in 5 cells (bottom left) and short duration response in 2 cells (bottom right).
(TIF)

S1 Movie Time series raster scan images of eGFP-EGFR^wt^-expressing CHO-K1 cell for RICS, before EGF addition. The focus was adjusted to the basal plasma membrane. Images were recorded with 20 μs pixel dwell time and 6.26 ms interline time. The original fluorescence image (left), the moving average of 10 consecutive frames (middle), and the image after the moving average subtraction (right) are shown. The intensity scale for original frames, moving average and the subtracted images is shown on the far right.
(MOV)

S2 Movie Time series raster scan images of eGFP-EGFR^wt^-expressing CHO-K1 cell for RICS, ~10 min after EGF addition. The imaging region and parameters are the same as for S1 Movie. Please note the round structures (endosomes) visible in the upper part of the moving average, and the absence of them in the final subtracted image.
(MOV)

S1 Text Detailed protocols. (DOCX)
