# Supplementary figures and images for: Inhibition of Receptor Dimerization as a Novel Negative Feedback Mechanism of EGFR Signaling
Source: PLoS One. 2015 Oct 14;10(10):e0139971. doi: 10.1371/journal.pone.0139971 (PMC4605717; doi:10.1371/journal.pone.0139971)

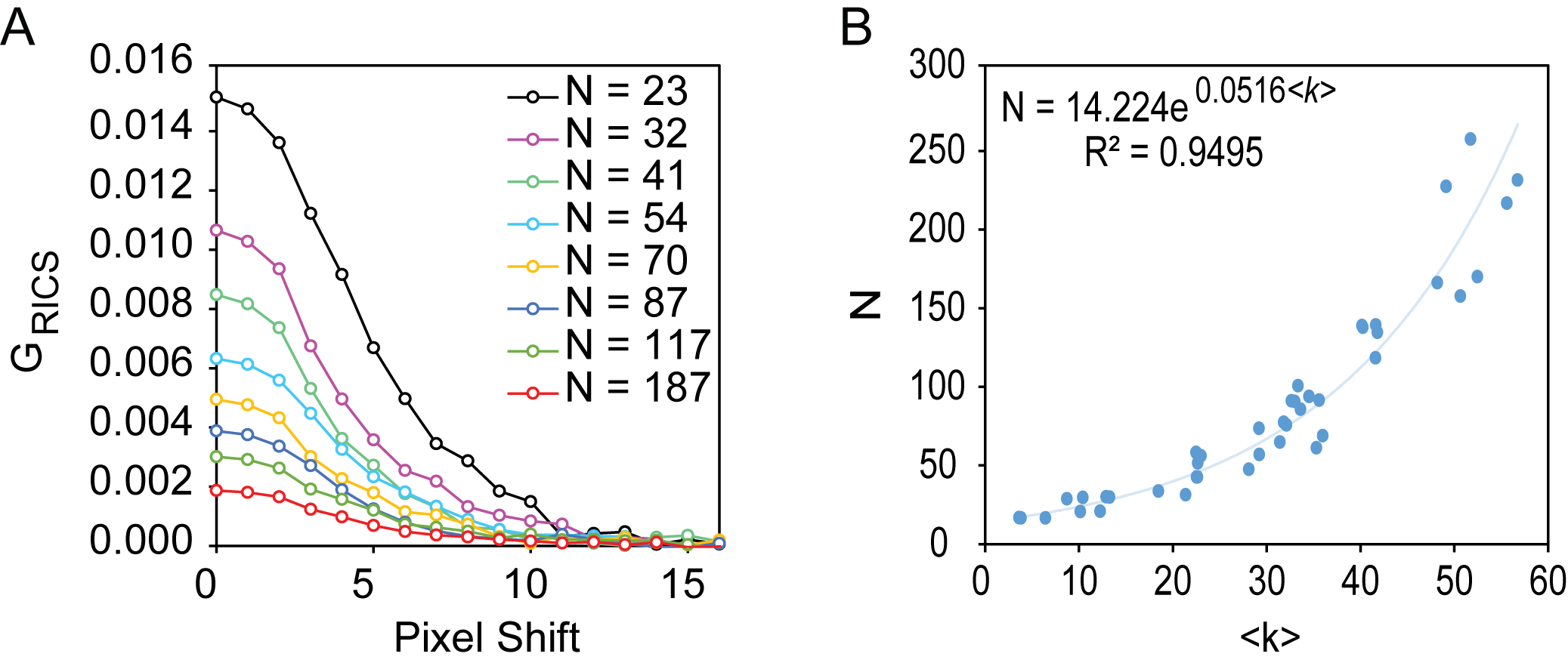

Supplement: S1 Fig — (A) Horizontal profiles of RICS autocorrelation amplitudes for eGFP solutions with different concentrations. Number of the molecules in the confocal volume (N) calculated using G(0,0) for respective curves is presented on the right. (B) The calibration curve showing the relationship between N and the fluorescence intensity. (TIF) [file pone.0139971.s001.tif]

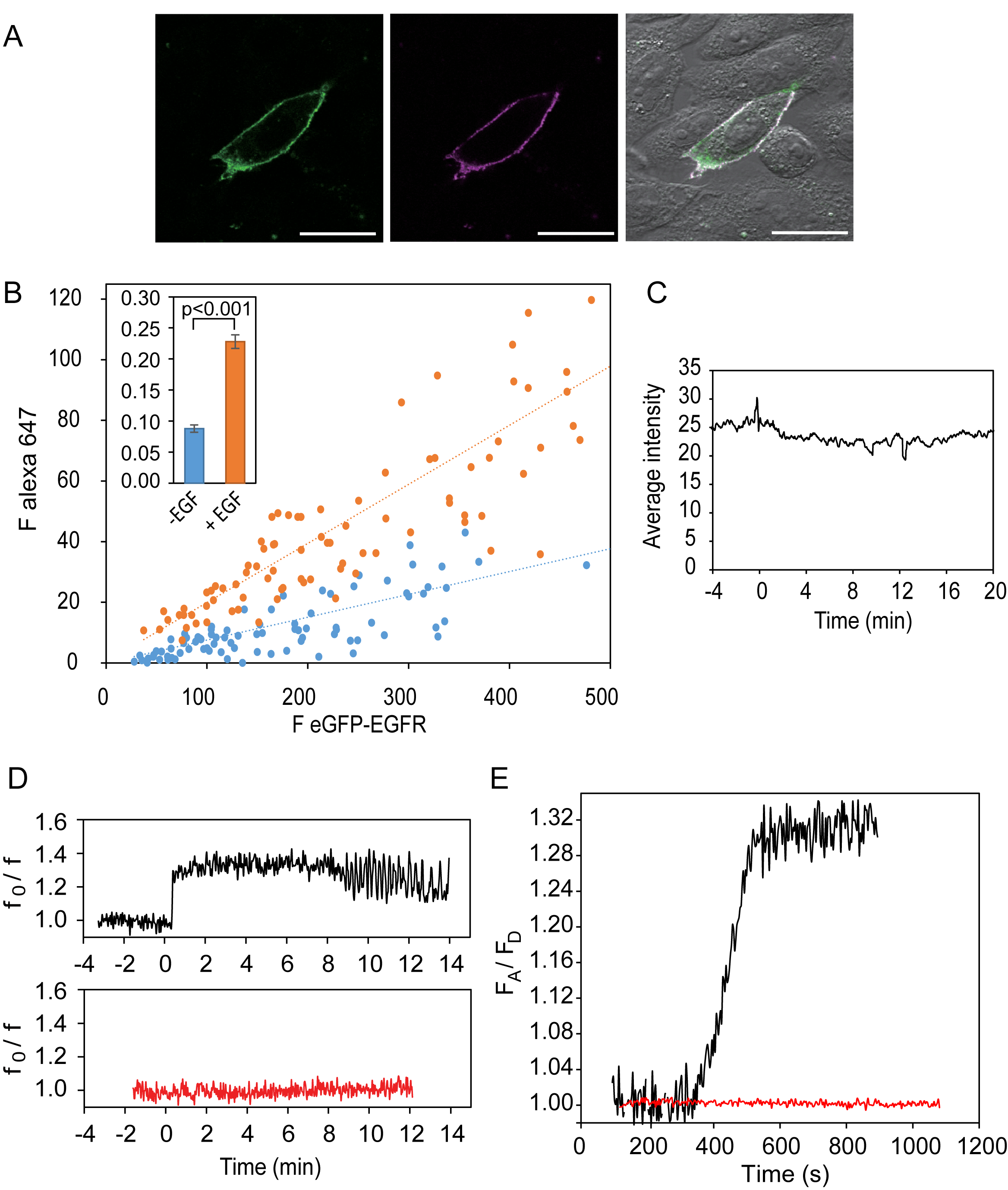

Supplement: S2 Fig — (A) EGF (Alexa647-labeled, 0.17 μM; magenta) binding to eGFP-EGFRwt (green) expressing cells but not to the control, non-transfected, cells seen on differential interference contrast image (DIC). Right panel shows both channels merged with a DIC image. Scale bars equal to 20 μm. (B) Phosphorylation of eGFP-EGFR at Y1173. Cells expressing eGFP-EGFRwt were immunostained using rabbit anti-human phospho-EGFR pY1173 antibody and Alexa647-labeled goat anti-rabbit IgG antibody. The plot shows fluorescence intensity of Alexa 647 versus eGFP for quiescent (blue) and EGF challenged (orange) cells. The slope of FAlexa 647 over FeGFP is presented in the inset (error bars indicate SEM). (C) Time trace of average fluorescence intensity of the eGFP-EGFRwt on the plasma membrane, at t = 0, EGF was added. (D) Cytosolic Ca2+ concentration monitored with Fura Red. Reproducible Ca2+ response in cells expressing eGFP-EGFRwt (black) and non-transfected control cells (red). (E) ERK activity monitored with EKAREV in CHO-K1 cells expressing eGFP-EGFRwt (black) and control cells (no eGFP-EGFRwt expression, red). We analyzed 53 and 66 cells (for eGFP-EGFRwt expressing and control cells, respectively) with essentially the same results. (TIF) [file pone.0139971.s002.tif]

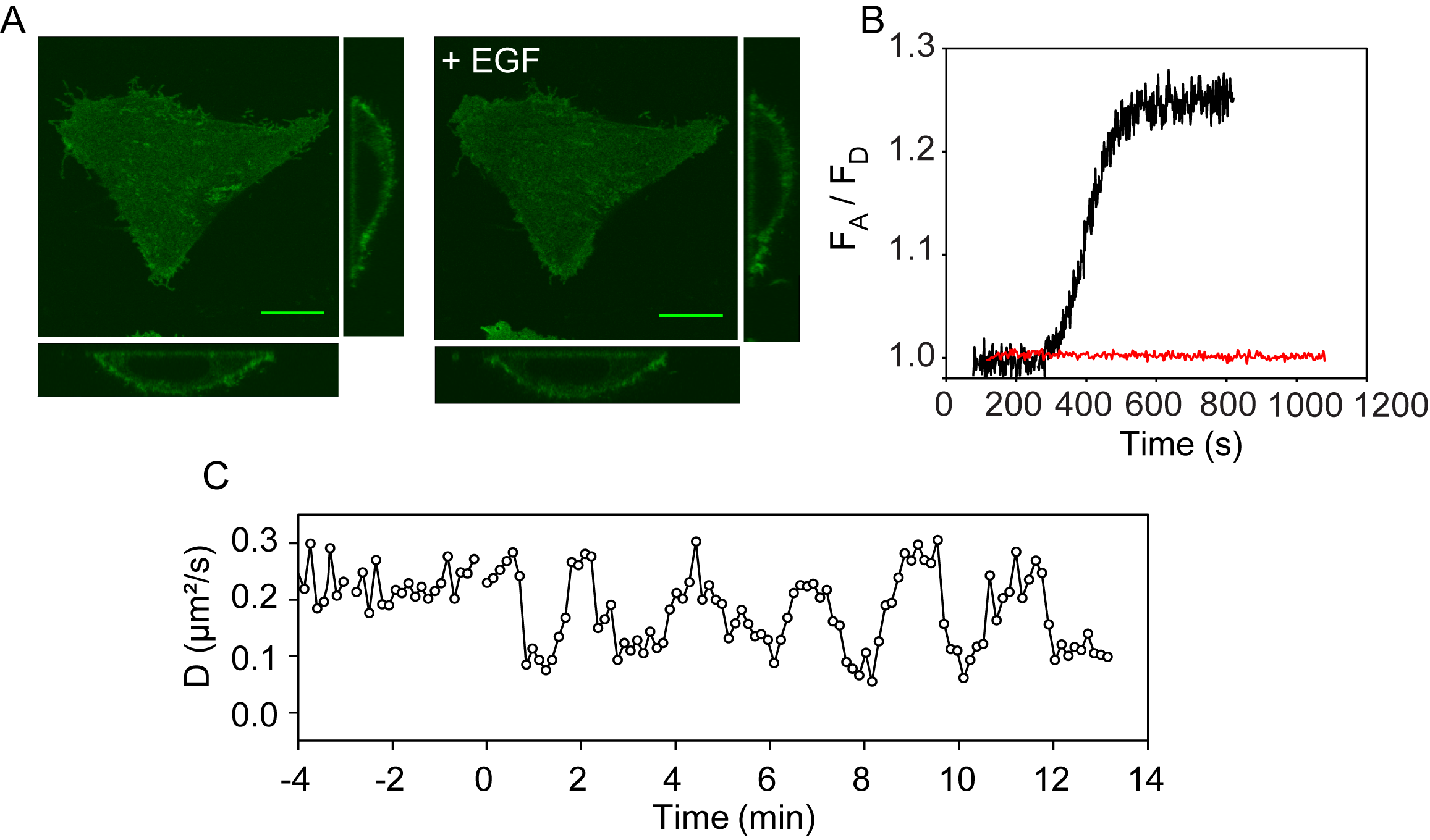

Supplement: S3 Fig — (A) Confocal images of the Z-projection and cross sections of eGFP-EGFRΔC995 in CHO-K1 cells before (left) and 30 min after EGF addition (right). After 30 min addition of EGF, eGFP-EGFRΔC995 was still exclusively targeted to the plasma membrane and no endosomes were observed. Scale bars indicate 10 μm. (B) ERK activity monitored with EKAREV in CHO-K1 cells expressing eGFP-EGFRΔC995 (black) and non-transfected control cells (red). (C) Reproducible time trace of eGFP-EGFRΔC995 diffusion coefficient. EGF (0.17 μM) was added at the t = 0. (TIF) [file pone.0139971.s003.tif]

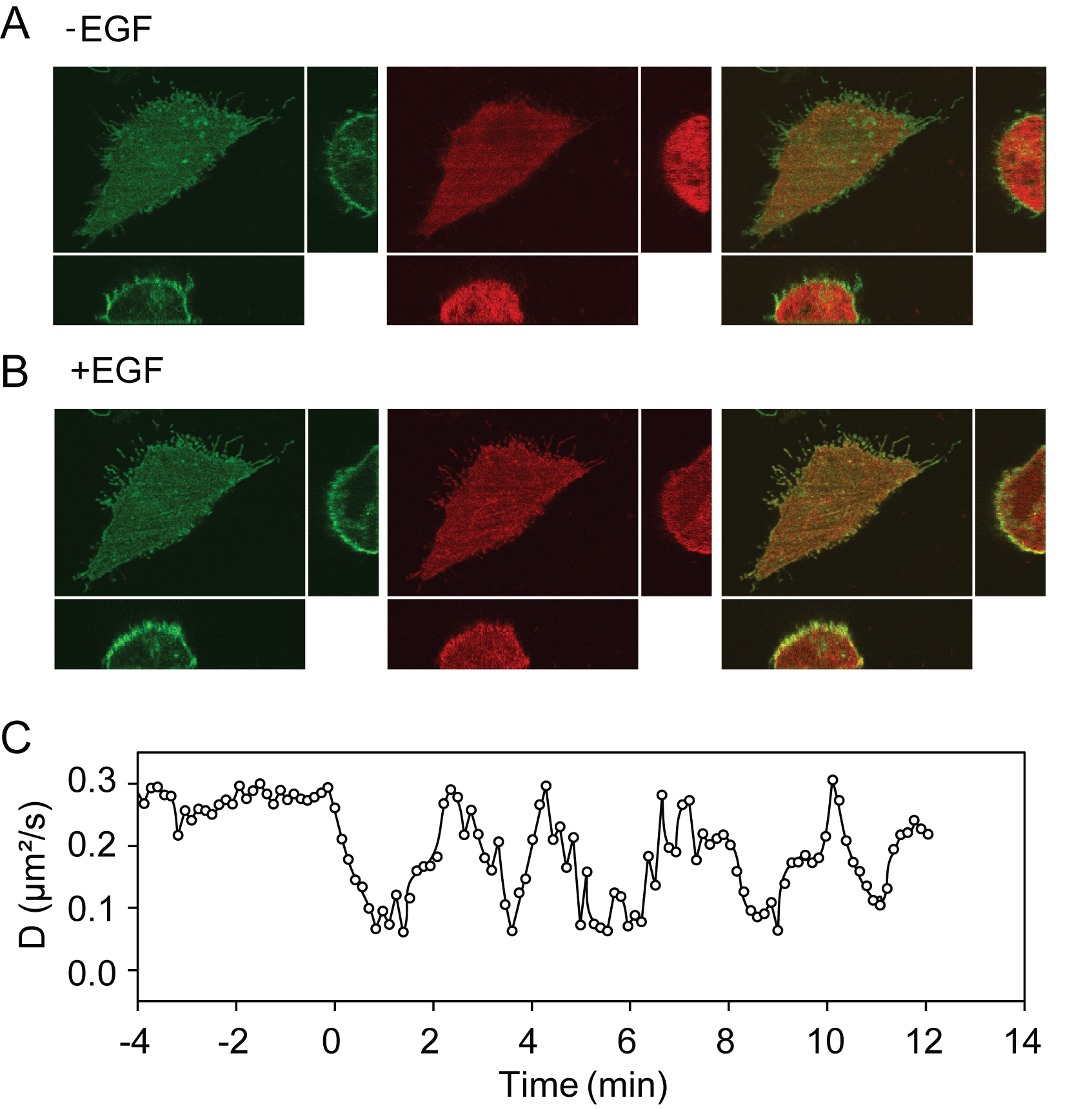

Supplement: S4 Fig — (A, B) Confocal images of the cells coexpressing eGFP-EGFRwt (green) and mCherry-PLCγ1wt (red) before (A) and immediately after EGF addition (B). Upon EGF challenging, the recruitment of mCherry-PLCγ1wt to the plasma membrane was observed, most probably through the binding to eGFP-EGFRwt. (C) The diffusion coefficient of eGFP-EGFRwt in the cell coexpressing mCherry-PLCγ1wt. (TIF) [file pone.0139971.s004.tif]

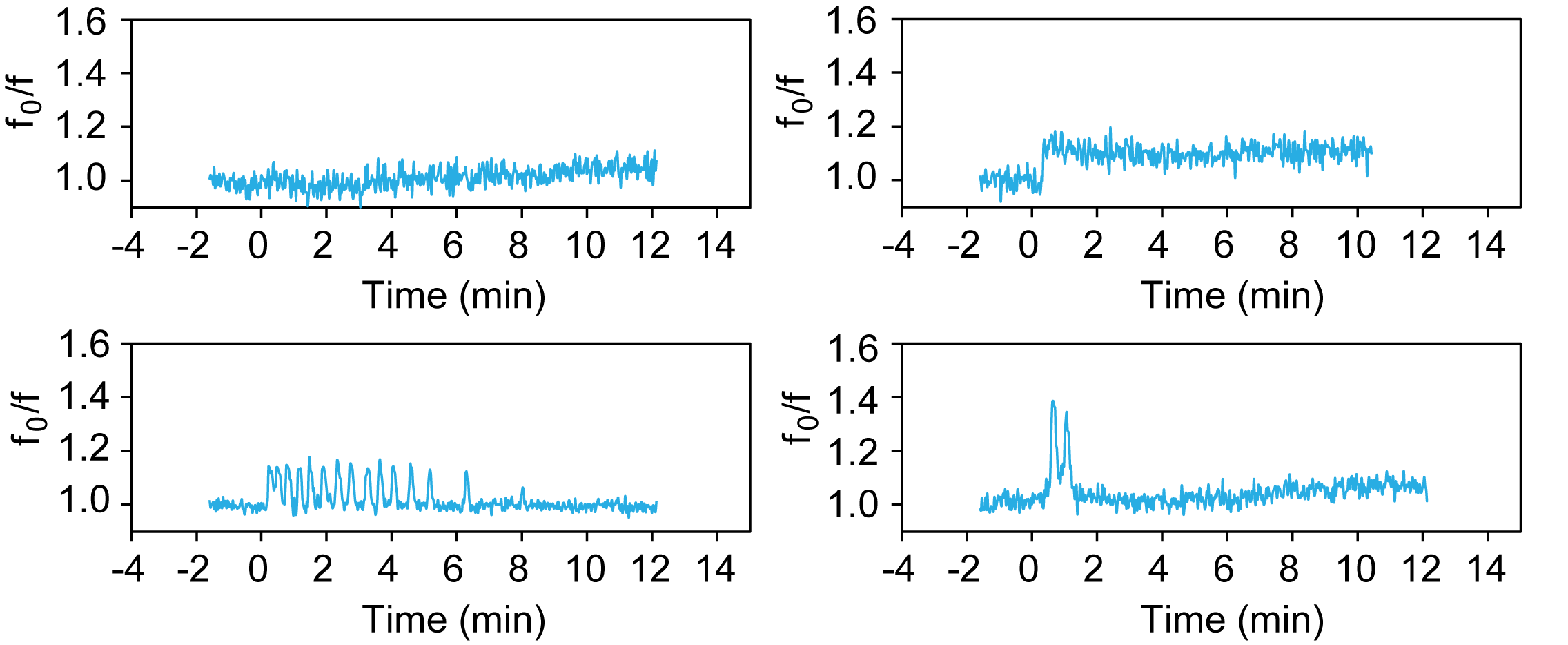

Supplement: S5 Fig — No Ca2+ response was observed in 31 out of 39 cells (upper left). Weak continuous response was observed in 1 cell (upper right), immediate oscillations with low amplitude in 5 cells (bottom left) and short duration response in 2 cells (bottom right). (TIF) [file pone.0139971.s005.tif]
