# Supplementary material for: Inhibition of Receptor Dimerization as a Novel Negative Feedback Mechanism of EGFR Signaling
Source: PLoS One. 2015 Oct 14;10(10):e0139971. doi: 10.1371/journal.pone.0139971 (PMC4605717; doi:10.1371/journal.pone.0139971)
Supplement: S1 Text — (DOCX) [file pone.0139971.s008.docx]

# Supporting Information

## Detailed protocols

### Plasmid construction

The gene encoding human EGF receptor (gift from Dr. Shibuya) was subcloned to a mammalian expression vector, pcDNA3 (Life Technologies, Carlsbad, CA), between the *HindIII/XbaI* sites. In order to insert eGFP after the sequence encoding signaling peptide (MRPSGTAGAALLALLAALCPASRA), the full-length plasmid of EGFR/pcDNA3 was amplified by PCR with a forward primer (5’-ATAAATAAGCGGCCGCTGAAAAGAAA-GTTTGCCAAGGCACG-3’) annealing to the EGFR sequence immediately after the signal sequence (ss) and reverse primer annealing to the end of the ss (5’-TTATTTAT-CTCGAGAGCCCGACTCGCCGGGC-3’). The primers contained additional *NotI* and *XhoI* sites, respectively. *XhoI* and *NotI* sites were also added to the N- and C-termini of the gene encoding eGFP by PCR using eGFP/pcDNA3 plasmid (gift from Dr. Miyawaki) as a template. After *XhoI*/*NotI* digestion, both fragments were ligated to obtain eGFP-EGFR/pcDNA3. A series of phosphomimic and phosphodeficient mutants of eGFP-EGFR (T654A, T654E, T669A, T669E, Y992F, Y1148F, and Y1173F) were made by site directed mutagenesis [1]. C-terminal truncated mutant of eGFP-EGFR^ΔC995^ was prepared by means of PCR with a reverse primer containing an *XbaI* site and an additional stop codon followed by the sequence annealing to just before the desired truncation site (5’- TAATTATTCTAGACTATGGGATGAGGTACTCGTCGGCATCCACCACG-3’). A forward primer used for this PCR was same as the one for the amplification of EGFR/pcDNA3. The PCR fragment was substituted for the *NotI/XbaI* fragment of eGFP-EGFR/pcDNA3.

The gene of human PLCγ1 was amplified from pCR-XL-TOPO plasmid (Clone ID: 9052656, Thermo Scientific, Rockford, IL). *EcoRI* and *NotI* restriction sites were added to N- and C-termini of PLCγ1 gene, respectively. *NotI* and *XbaI* restriction sites were added to the gene encoding mCherry by PCR using mCherry/pRSET plasmid (gift from Dr. Tsien) as a template. Both PCR fragments were subcloned to *EcoRI/XbaI* sites of pcDNA3 vector yielding PLCγ1-mCherry/pcDNA3 plasmid. The phosphomimic mutant of PLCγ1, Y783F, was made by the site directed mutagenesis.

The restriction fragments of HA-tagged human PDK1-K612W and S738E/S742E were prepared by cutting HA.PKD.K/W [2] and HA.PKD.S738E/S742E [3], respectively, (gift from Dr. Toker, Addgene: 10810 and 10809) with *BamHI* and *XhoI*. *HindIII* and *BamHI* sites were added to the N- and C-terminals of the mCherry gene by PCR. The *HindIII*-mCherry-*BamHI* and *BamHI*-HA.PKD.K/W-*XhoI* or *BamHI*-HA.PKD.S738E/S742E-*XhoI* fragments were subcloned to *HindIII/XhoI* sites of pcDNA3 to acquire plasmids to express mCherry-PDK1 (mCherry-HA-PKD1-K612W-pcDNA3 and mCherry-HA-PKD1-S738E/S742E-pcDNA3, respectively).

A plasmid to express plasma membrane targeted mCherry was constructed by attaching a gene of the N-terminal chain of non-receptor tyrosine kinase (Lyn), which contains palmitoylation and myristoylation sites anchoring the fusion protein to the plasma membrane. The gene encoding the N-terminal chain of Lyn (ATGGGCTGCAT-CAAGAGCAAGCGCAAGGACAACCTGAACGACGACGGCGTGGAC) was amplified using primers containing HindIII and XhoI overhangs (forward primer: TATATATAAAGC-TTATGGGCTGCATCAAGAGCAAGCGCAAGGACAACCTGAACGACGACG and reverse primer: TATATTATCTCGAGGTCCACGCCGTCGTCGTTCAGGTTGTCCTTCCGTTTGGACTTGAT). XhoI and XbaI sites were added by PCR to the genes encoding eGFP and mCherry respectively. The respective fragments were subcloned on pcDNA3 to obtain Lyn-eGFP/pcDNA3 and Lyn-mCherry/pcDNA3.

### Raster image correlation spectroscopy (RICS)

The RICS auto-correlation function of 2D membrane bound diffusion model $G_{RICS}\left( \xi,\psi\right)$ is described by the spatial component $\left( \xi,\psi\right)$ reflecting the movement of the scanning laser beam $S\left( \xi,\psi\right)$ and the auto-correlation decay due to diffusion of the molecules$G\left( \xi,\psi\right)$:

$G_{RICS}\left( \xi,\psi\right)=S\left( \xi,\psi\right)\cdot G\left( \xi,\psi\right)$ (1)

$S\left( \xi,\psi\right)=exp\left( -\frac{\left( \frac{\left| \xi\right|\delta x}{w_{0}} \right)^{2}+\left( \frac{\left| \psi\right|\delta y}{w_{0}} \right)^{2}}{\left( 1+\frac{4D\tau_{p}\left| \xi\right|+\tau_{l}\left| \psi\right|}{w_{0}} \right)} \right)$ (2)

$G\left( \xi,\psi\right)=\frac{\gamma}{N}\left( 1+\frac{4D\left( \tau_{p}\left| \xi\right|+\tau_{l}\left| \psi\right| \right)}{{w_{0}}^{2}} \right)^{-1}$ (3)

where *δx* and *δy* is the pixel size, *τ_p_* and *τ_l_* are pixel dwell time and interline time, respectively. The illumination of focal volume was assumed to be an ideal 3D Gaussian resulting in the shape factor of γ = 0.3535 [4]. The radius of the focal volume, *w*_0_, was determined by use of 100 - 200 image stacks of recombinant eGFP [5] in water solution (*D* = 95 µm²/s) [6-7] acquired using the same experimental setup with matching scanning speed (12.5 µs/pixel).

The RICS auto-correlation is calculated by multiplying one matrix corresponding to the original image by the matrix corresponding to the image shifted in the x and y directions [8]. In the case of cross-correlation RICS (ccRICS) [9], both original and shifted images come from two separate detection channels (e.g. eGFP and mCherry). Cross-correlation of the signal from two separate detection channels indicates an interaction between fluorescent species detectable in these channels. The cross-correlation amplitude [*G(0,0)_cc_*] is related to the number of interacting molecules.

### Number and brightness (N&B) analysis

Each pixel contains information over the fluorescence intensity $k_{i}$ and following this intensity over $K$ number of frames allows for calculating its average intensity $\left\langle k \right\rangle$. $\left\langle k \right\rangle$ of particular pixel depends on average number of molecules $n$ (in N&B analysis both EGFR monomer and dimer are regarded as single molecules) and their true molecular brightness *ε* (counts/pixel dwell time/molecule) [10-11]:

$\left\langle k \right\rangle=\frac{\sum_{i} k_{i}}{K}= \varepsilon n$ (4)

For respective pixels, $\varepsilon$ is determinable due to their different distributions of fluorescence intensity fluctuations (the variance, $\sigma^{2}$):

$\sigma^{2}=\frac{\sum_{i} \left( k_{i}-\left\langle k \right\rangle\right)^{2}}{K}$ (5)

Total variance of the system equals to the sum of $\sigma_{n}^{2}$ – variance due to the number fluctuations, which depends on the square of particle brightness, and $\sigma_{0}^{2}$ – the detector variance which equals to the intensity:

$\sigma_{n}^{2}=\varepsilon^{2}n$ (6)

$\sigma_{0}^{2}=\varepsilon n$ (7)

$\sigma^{2}=\sigma_{n}^{2}+\sigma_{0}^{2}$ (8)

Apparent molecular brightness *B* – ratio of variance to average intensity is directly related to *ε* and independent of *n*. The apparent molecular brightness gives rise to assess *ε* of particular species in each pixel of scanned image stack:

$B=\frac{\sigma^{2}}{\left\langle k \right\rangle}=\frac{\sigma_{n}^{2}}{\left\langle k \right\rangle}+\frac{\sigma_{0}^{2}}{\left\langle k \right\rangle}=\frac{\varepsilon^{2}n}{\varepsilon n}+\frac{\varepsilon n}{\varepsilon n}=\varepsilon+1$ (9)

Because of using the photomultiplier tube detector in photon counting mode, the distribution of digital levels (S) and the detector offset had to be calibrated. The S parameter, representing the apparent molecular brightness of immobile fraction (B = 1), was determined by acquiring images of a fluorescent solid slide that has no fluctuations. The offset was determined by acquiring a stack of images with the laser turned off. In our calculations a 3x3 median filter was applied to a stack of images subjected for analysis to delete large isolated fluctuations.

### Singe cell calculations of eGFP-EGFR expression level and mobile fraction

Based on the fluorescence intensity, the expression level of eGFP-EGFR was determined in respective cells. For calibration, 50 sequential raster images were acquired for recombinant eGFP solution [5] at various concentrations. The imaging parameters were same as for the eGFP-EGFR expressed in CHO-K1 cells except the pixel dwell time; the pixel dwell time (12.5 µs instead of 20 µs). The images were subjected for the RICS analysis. The radius of the confocal volume, *w*_0_, was determined to 0.24 ± 0.03 µm by fitting the calculated auto-correlation to a 3D diffusion model [12], with fixed diffusion coefficient (D = 95 µm²/s) [6] [7]. The number of molecules in the confocal volume $N$ was calculated from the auto-correlation amplitude,$G\left( 0,0 \right)$:

$G\left( 0,0 \right)=\frac{\gamma}{N}$ (10)

The illumination of focal volume was assumed to be an ideal 3D Gaussian resulting in the shape factor of $\gamma$ = 0.3535 [4]. The fluorescence intensity is proportional to the pixel dwell time. Average fluorescence intensity of 50 images was calculated and corrected for the different pixel dwell time by multiplying 20/12.5 ($<k>$). $N$ versus $<k>$ was fitted to an exponential model

$N=14.2249e^{0.0516<k>}$ (11)

with R² > 0.94 (Fig. S1B). This calibration curve was used to calculate the number of the molecules in the confocal volume for the cells expressing eGFP-labeled EGFR (*wt* and mutants). Because eGFP-EGFR was targeted exclusively to the plasma membrane in the measured cells, we could calculate the density of the receptors from *w*_0_ and *N*. Surface area of the basal plasma membrane of eGFP-EGFR transfected cells was calculated by using Fiji [13].

The fraction of mobile eGFP-EGFR molecules on the plasma membrane of the eGFP-EGFR^wt^ expressing CHO-K1 cells was calculated using fluorescence recovery after photobleaching (FRAP) [14]. For each FRAP experiment the eGFP-EGFR^wt^ fluorescence intensity was recorded in three equally sized regions of interest: the recovery region, the reference spot and the background, respectively. The background region was recorded outside the cell. The fluorescence of the reference spot, positioned at similar average fluorescence intensity as the recovery region in the basal plasma membrane of the same cell, was used to account for acquisition bleaching. Each FRAP experiment was proceeded by acquiring 5 s long fluorescence intensity time trace of all three regions, using 488-nm laser at 4% of the power with the optical settings as for confocal image acquisition. The recovery region was subsequently bleached for 100 ms using 488-nm laser at 100% of the power. After the bleaching, the fluorescence intensity of all three regions was acquired for 70 seconds. To account for acquisition bleaching, the recorded fluorescence intensity was normalized using equation:

$I_{N}\left( t \right)=\frac{I(t)\left\langle R_{p} \right\rangle}{\left\langle I_{p} \right\rangle R(t)}$ (12)

with the background-subtracted intensity of the recovery region *I(t)* and the reference spot $R(t)$, respectively. $\left\langle I_{p} \right\rangle$ and $\left\langle R_{p} \right\rangle$ are the pre-bleaching average intensities of the recovery region and the refererence spot, respectively. The mobile fraction of the eGFP-EGFR^wt^ was estimated with following equation:

$F_{mobile}=\frac{I_{final}-I_{0}}{\left\langle I_{p} \right\rangle-I_{0}}$ (13)

with *I_final_* being the end value of the normalized recovered fluorescence intensity *I_N_(t)*, and *I_0_* the first post-bleach intensity value acquired.

# References

1. Sawano A, Miyawaki A (2000) Directed evolution of green fluorescent protein by a new versatile PCR strategy for site-directed and semi-random mutagenesis. Nucleic Acids Res 28: E78.

2. Storz P, Döppler H, Johannes F-J, Toker A (2003) Tyrosine phosphorylation of protein kinase D in the pleckstrin homology domain leads to activation. J Biol Chem 278: 17969–17976.

3. Storz P, Döppler H, Toker A (2004) Protein kinase Cdelta selectively regulates protein kinase D-dependent activation of NF-kappaB in oxidative stress signaling. Mol Cell Biol 24: 2614–2626.

4. Brown CM, Dalal RB, Hebert B, Digman MA, Horwitz AR, et al. (2008) Raster image correlation spectroscopy (RICS) for measuring fast protein dynamics and concentrations with a commercial laser scanning confocal microscope. J Microsc 229: 78–91.

5. Heim R, Cubitt AB, Tsien RY (1995) Improved green fluorescence. Nature 373: 663–664.

6. Schenk A, Ivanchenko S, Röcker C, Wiedenmann J, Nienhaus GU (2004) Photodynamics of red fluorescent proteins studied by fluorescence correlation spectroscopy. Biophys J 86: 384–394.

7. Petrásek Z, Schwille P (2008) Precise Measurement of Diffusion Coefficients using Scanning Fluorescence Correlation Spectroscopy. Biophys J 94: 1437.

8. Digman MA, Brown CM, Sengupta P, Wiseman PW, Horwitz AR, et al. (2005) Measuring fast dynamics in solutions and cells with a laser scanning microscope. Biophys J 89: 1317–1327.

9. Digman MA, Wiseman PW, Horwitz AR, Gratton E (2009) Detecting protein complexes in living cells from laser scanning confocal image sequences by the cross correlation raster image spectroscopy method. Biophys J 96: 707–716.

10. Digman MA, Dalal R, Horwitz AF, Gratton E (2008) Mapping the number of molecules and brightness in the laser scanning microscope. Biophys J 94: 2320–2332.

11. Digman MA, Wiseman PW, Choi C, Horwitz AR, Gratton E (2009) Stoichiometry of molecular complexes at adhesions in living cells. Proc Natl Acad Sci USA 106: 2175.

12. Digman MA, Sengupta P, Wiseman PW, Brown CM, Horwitz AR, et al. (2005) Fluctuation correlation spectroscopy with a laser-scanning microscope: exploiting the hidden time structure. Biophys J 88: L33–L36.

13. Schindelin J, Arganda-Carreras I, Frise E, Kaynig V, Longair M, et al. (2012) Fiji: an open-source platform for biological-image analysis. Nat Methods 9: 676–682.

14. Axelrod D, Koppel DE, Schlessinger J, Elson E, Webb WW (1976) Mobility measurement by analysis of fluorescence photobleaching recovery kinetics. Biophys J 16: 1055–1069.
